# *GbCYP86A1‐1* from *Gossypium barbadense* positively regulates defence against *Verticillium dahliae* by cell wall modification and activation of immune pathways

**DOI:** 10.1111/pbi.13190

**Published:** 2019-06-26

**Authors:** Guilin Wang, Jun Xu, Lechen Li, Zhan Guo, Qingxin Si, Guozhong Zhu, Xinyu Wang, Wangzhen Guo

**Affiliations:** ^1^ State Key Laboratory of Crop Genetics & Germplasm Enhancement Nanjing Agricultural University Nanjing Jiangsu Province China; ^2^ College of Life Sciences Nanjing Agricultural University Nanjing Jiangsu Province China

**Keywords:** cytochrome P450 fatty acid ω‐hydroxylase, *Verticillium dahliae*, *GbCYP86A1‐1*, *Gossypium barbadense*, cell wall modification, immune pathway

## Abstract

Suberin acts as stress‐induced antipathogen barrier in the root cell wall. CYP86A1 encodes cytochrome P450 fatty acid ω‐hydroxylase, which has been reported to be a key enzyme for suberin biosynthesis; however, its role in resistance to fungi and the mechanisms related to immune responses remain unknown. Here, we identified a disease resistance‐related gene, *GbCYP86A1‐1*, from *Gossypium barbadense* cv. Hai7124. There were three homologs of *GbCYP86A1* in cotton, which are specifically expressed in roots and induced by *Verticillium dahliae*. Among them, *GbCYP86A1‐1* contributed the most significantly to resistance. Silencing of *GbCYP86A1‐1* in Hai7124 resulted in severely compromised resistance to *V. dahliae*, while heterologous overexpression of *GbCYP86A1‐1* in *Arabidopsis* improved tolerance. Tissue sections showed that the roots of *GbCYP86A1‐1* transgenic *Arabidopsis* had more suberin accumulation and significantly higher C16‐C18 fatty acid content than control. Transcriptome analysis revealed that overexpression of *GbCYP86A1‐1* not only affected lipid biosynthesis in roots, but also activated the disease‐resistant immune pathway; genes encoding the receptor‐like kinases (RLKs), receptor‐like proteins (RLPs), hormone‐related transcription factors, and pathogenesis‐related protein genes (PRs) were more highly expressed in the *GbCYP86A1‐1* transgenic line than control. Furthermore, we found that when comparing *V. dahliae* ‐inoculated and noninoculated plants, few differential genes related to disease immunity were detected in the *GbCYP86A1‐1* transgenic line; however, a large number of resistance genes were activated in the control. This study highlights the role of *GbCYP86A1‐1* in the defence against fungi and its underlying molecular immune mechanisms in this process.

## Introduction

*Verticillium dahliae* is a soil‐borne fungal pathogen that can infect up to 200 plant species and poses a destructive threat to crops (Fradin and Thomma, 2006). Signals from plant roots trigger the life cycle of *V. dahliae*, beginning with the germination of microsclerotia (Klimes *et al*., 2015). The germinated hyphae penetrate the epidermal cells of the roots and then colonize the xylem vessels (Zhao *et al*., 2014). After successful colonization, the fungus continues to grow along the vascular tissues and produce conidia and microsclerotia, which block water transport or xylem vessels, eventually leading to plant wilting or defoliation (Klimes *et al*., 2015). Due to the limited number of resistance genes, low effective control measures and few high resistance germplasm resources to cope with *V. dahliae*, it is difficult to control once plants are infected with the disease (Klosterman *et al*., 2009).

The most important factor in preventing the invasion of *V. dahliae* from roots is the epidermal cell wall, which provides a mechanical and chemical barrier as the first line of defence, including a suberin lamella, thick cuticle and synthesis of phenolic compounds (Schreiber, 2010). In addition, plants activate a complex series of immune pathways to delay or block further expansion of the pathogen inside the cell (Jones and Dangl, 2006). Pattern recognition receptors (PRRs) on the surface of the plasma membrane sense pathogen‐associated molecular patterns (PAMPs) and damage‐related molecular patterns (DAMPs), which could cause the response of internal defence‐associated phytohormones, activation of pathogenesis‐related genes (PRs) and production of reactive oxygen species (ROS) (Álvarez and Vasquez, 2017; Bacete *et al*., 2018). PRRs are either surface‐localized receptor‐like kinases (RLKs) or receptor‐like proteins (RLPs) containing various ligand‐binding ecto‐domains that perceive PAMPs or DAMPs (Gómez‐Gómez and Boller, 2000; Zipfel, 2014). A typical DAMP is represented by oligogalacturonides (OGs), which are the main component of cell wall pectin. OGs can be released by polygalacturonase‐inhibiting proteins (PGIPs) and pectate lyases in the early stage of pathogen infection (Benedetti *et al*., 2015; Brutus *et al*., 2010). As a powerful dynamic barrier, plant cell walls can trigger a variety of changes in response to pathogen attack. Some of these are well described, but many remain obscure (Malinovsky *et al*., 2014; Nühse, 2012).

Plants have special phenolic ester‐based protection system that is accompanied by various metabolic and structural adaptations, including the formation of cell wall modifications, such as the above‐ground cuticles and the wide distributed suberin in root (Kolattukudy, 1980; Pollard *et al*., 2008). Suberin is a glycerolipid–phenolic polymer with a sturdy structure and the chemical properties of a hydrophobic polymer, which make the root resistant to pathogen infection. Histochemical studies have demonstrated that environmental stress can enhance the suberin's production (Franke and Schreiber, 2007). For example, treatment with *V. dahliae* or stress‐responsive hormones induces suberin accumulation in tomato cell wall (Robb *et al*., 1991). When the tuber of potato is damaged, it can induce the suberization of peridermis and increase the resistance to the *V. dahliae* (Lulai, 2005). Thangavel *et al*. (2016) screened potato somatic cells that were resistant to *Streptomyces* spp toxin and found that there was higher expression of genes associated with suberin biosynthesis, indicating the important role of suberin in the resistance of potato to pathogens. In addition, cell wall suberization can also increase the resistance of *Glycine max* to *Phytophthora sojae*, thus avoiding decay of roots and stems (Thomas *et al*., 2007). In *Arabidopsi*s, it has also been found that the invasion of pathogens induces cell wall suberization (Franke *et al*., 2005). Therefore, the suberized cell walls are more conducive to the defence against pathogen infection than nonmodified cell walls composed of carbohydrates (Franke and Schreiber, 2007).

CYP86 is a subfamily of the cytochrome P450 (CYP) family that is mainly involved in the hydroxylation of fatty acyl‐CoA ω sites to form ω‐hydroxy acids, some of which are oxidized to α,ω‐dicarboxylic acids (Werck‐Reichhart and Feyereisen, 2000), and further involved in the biosynthesis of protective biopolymers such as cutin and suberin (Kandel *et al*., 2006). *AtCYP86A2*,* AtCYP86A4* and *AtCYP86A8* are three homologous genes with high similarity that play a role in the biosynthesis of extracellular lipids and are involved in the hydroxylation of long‐chain fatty acids. Previous studies have indicated that these genes are involved in the process of cell wall cuticle modification, and the certain cutin‐related fatty acids synthesized by *AtCYP86A2* may inhibit bacterial type III gene expression (Wellesen *et al*., 2001; Xiao *et al*., 2004). *AtCYP86A1*, which is involved in the hydroxylation of C16‐C18 long‐chain fatty acids, was the first fatty acid ω‐hydroxylase identified in plants (Benveniste *et al*., 1998). Several studies have identified that *AtCYP86A1* is a key enzyme for aliphatic root suberin biosynthesis. *Arabidopsis CYP86A1* mutants showed a significant decrease in C16 and C18 suberin monomer content in roots, and the total aliphatic suberin content decreased by 60%. Simultaneously, *CYP86A1* has been localized to the endoplasmic reticulum (ER) of root endodermis cells, indicating that suberin monomer biosynthesis takes place in this subcellular compartment before intermediates are exported in the cell wall (Höfer *et al*., 2008). After RNA interference with *StCYP86A1*, the content of C16 and C18 ω‐hydroxy acid and α, ω‐dicarboxylic acid decreased by about 70–90% (Serra *et al*., 2009). *CYP86B1* is clustered in a different class to *CYP86A1*. As a very long‐chain fatty acid hydroxylase, CYP86B1 participates in the hydroxylation of C22‐C24 very long‐chain fatty acids (Compagnon *et al*., 2009). Compared to wild type (WT), knockout of *AtCYP86B1* led to almost complete lack of C22 and C24 corresponding α,ω‐dicarboxylic acids and ω‐hydroxy acids in root and seed coat aliphatic suberin (Molina *et al*., 2009). Overall, CYP86 genes encoding fatty acid hydroxylase are inextricably linked to protective biopolymer biosynthesis (Duan and Schuler, 2005; Höfer *et al*., 2008).

As one of the world's major cash crops, cotton is the most important source of textile fibre and seed oil, but damage from *V. dahliae* seriously affects the yield and quality (Daayf *et al*., 1997). Studying the biosynthesis of suberin in roots has the potential to increase the defence against *V. dahliae* colonization and invasion (Franke and Schreiber, 2007). Previous studies have reported that the CYP86 subfamily genes participate in the synthesis of suberin monomers; however, the related function and application in improving disease resistance to *V. dahliae* remain largely unknown. With the release of whole‐genome data based on different *Gossypium* species, including *G. raimondii* (D5), *G. arboreum* (A2), *G. barbadense* cv. Hai7124 (AD2) and *G. hirsutum* acc, TM‐1 (AD1) (Hu *et al*., 2019; Li *et al*., 2014; Paterson *et al*., 2012; Zhang *et al*., 2015), in this study, we systematically surveyed CYP86 subfamily genes in these four cotton species and analysed their structure and expression characteristics. We found that there were three homologous genes of CYP86A1 in cotton. They were all specifically expressed in roots and were highly expressed in response to *V. dahliae* challenge. Silencing of each of three genes in disease‐resistant *G. barbadense* cv. Hai7124 significantly impaired resistance to *V. dahliae*, while overexpression of each in *Arabidopsis* increased disease resistance. Of them, *GbCYP86A1‐1* showed the most significant resistance phenotype. Further studies indicate that overexpression of *GbCYP86A1‐1* not only improved structural resistance in roots through greater accumulation of suberin, but also activated the disease resistance‐related immune pathways. This is the first report that *GbCYP86A1‐1* plays an important role in *V. dahliae* resistance, and we also provide effective gene resources for the development of *Verticillium* wilt‐resistant cultivars through cotton breeding programmes.

## Results

### Identification and expression patterns of the CYP86 genes in *Gossypium*

Based on P450 domain and whole‐genome sequence of *Gossypium raimondii,* we identified 373 GrCYPs. Through phylogenetic analysis with 245 AtCYPs in *Arabidopsis* (Xu *et al*., 2015), ten GrCYP86 genes were further identified (Figure S1). Following homology alignment analysis, we identified 10, 19 and 19 CYP86s in *G. arboreum*,* G. hirsutum* acc. TM‐1 and *G. barbadense* cv. Hai7124, respectively, and carried out their systematic naming based on the corresponding homology in *Arabidopsis* (Data S1). GrCYP86s can be divided into three classes (A, B, and C) (Figure S2a). There was more than 80% homology between homologous genes of the same class, with the exception of GrCYP86B1 (Figure S2b; Table S1). Only three genes, *GrCYP86A7‐2*,* GrCYP86B1‐1* and *GrCYP86B1‐2*, had introns (Figure S2c). All GrCYP86s had a P450 domain, including the helix K domain, helix I domain and heme‐binding domain (Figure S3), and a transmembrane domain at the N terminus, with the exception of GrCYP86A7s (Figure S2d).

**Figure 1:**
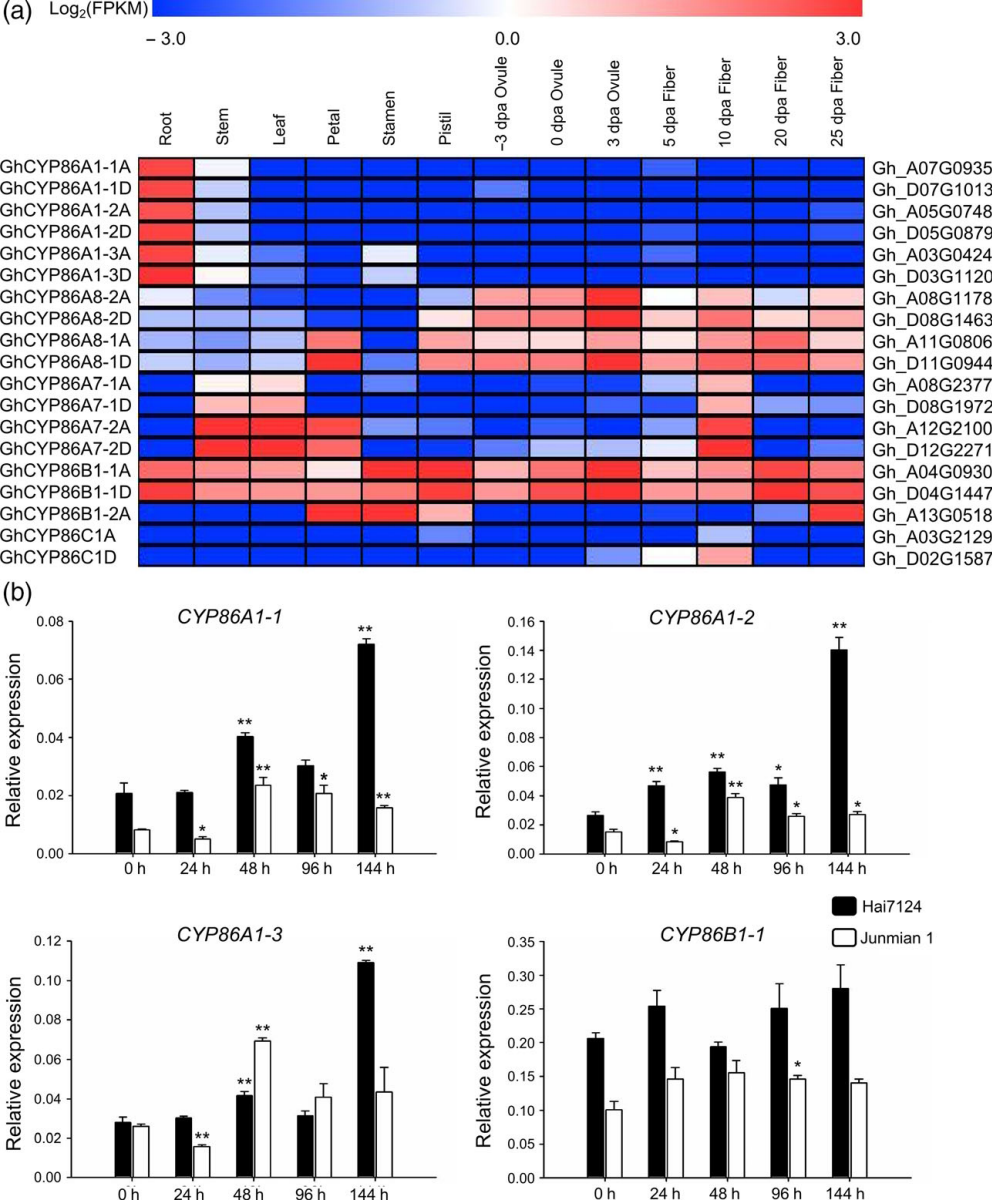
Expression patterns of CYP86 genes in different tissues and upon *Verticillium dahliae* challenge in cotton. (a) Transcriptional profiling of CYP86 genes in different tissues and organs in *G. hirsutum* acc. TM‐1. Root, stem, leaf, petal, stamen, ovule at ‐3, 0 and 3 dpa, fibre at 5, 10, 20 and 25 dpa were used for the comparative transcriptome analysis. The expression data were converted to log_2_ (FPKM) to calculate the expression levels of the CYP86 genes. Coloured squares indicated expression levels from ‐3 (blue) to 3 (red). The RNA‐seq data used here could be downloaded from http://www.ncbi.nlm.nih.gov/bioproject/ PRJNA248163/. (b) Expression patterns of CYP86 genes in response to *V. dahliae* infection were analysed by qRT–PCR in the resistant cultivar *G. barbadense* cv. Hai7124 and the susceptible cultivar *G. hirsutum* cv. Junmian 1, respectively. Error bars represent the standard deviation of three biological replicates. The statistical analyses were performed by comparing expression levels at different time points after *V. dahliae* infection to 0 h without inoculation using Student's *t*‐test (*P < 0.05, **P < 0.01), respectively.

Transcriptome data from TM‐1 vegetative tissues (root, stem and leaf), floral tissues (petal, stamen and pistil), ovule tissues and fibre tissues at different developmental stages showed that *GhCYP86s* had diverse developmental and spatial regulation in various tissues, with similar expression patterns in the homologous genes (Figure 1a; Data S2). The *GhCYP86A1* homologs were specifically expressed in roots. *GhCYP86A8* homologs were predominantly expressed in reproductive organs. *GhCYP86A7* homologs were expressed in stem, leaf, petal and 10 dpa fibre tissues. *GhCYP86B1‐1* was highly expressed in all tissues, but *GhCYP86B1‐2* was only expressed in floral and 25 dpa fibre tissues. *GhCYP86C1* was not expressed in most tissues. These findings indicate that CYP86s have conserved structures but diverse functions.

With detectable expression levels in root tissues following transcriptome analysis, the responses of *CYP86A1‐1*,* CYP86A1‐2*,* CYP86A1‐3* and *CYP86B1‐1* were further investigated after *V. dahliae* inoculation in cotton. Overall, the expression of these *CYP86s* was higher in *G. barbadense* cv. Hai7124 than *G. hirsutum* cv. Junmian 1. In particular, the expression of *CYP86A1‐1*,* CYP86A1‐2* and *CYP86A1‐3* was significantly increased after inoculation in these two plants, especially in Hai7124 after 144 h (Figure 1b). Therefore, these three genes are related to *V. dahliae* resistance.

### Functional characterization of GbCYP86A1s in *V. dahliae* resistance through virus‐induced gene silencing (VIGS)

**Figure 2:**
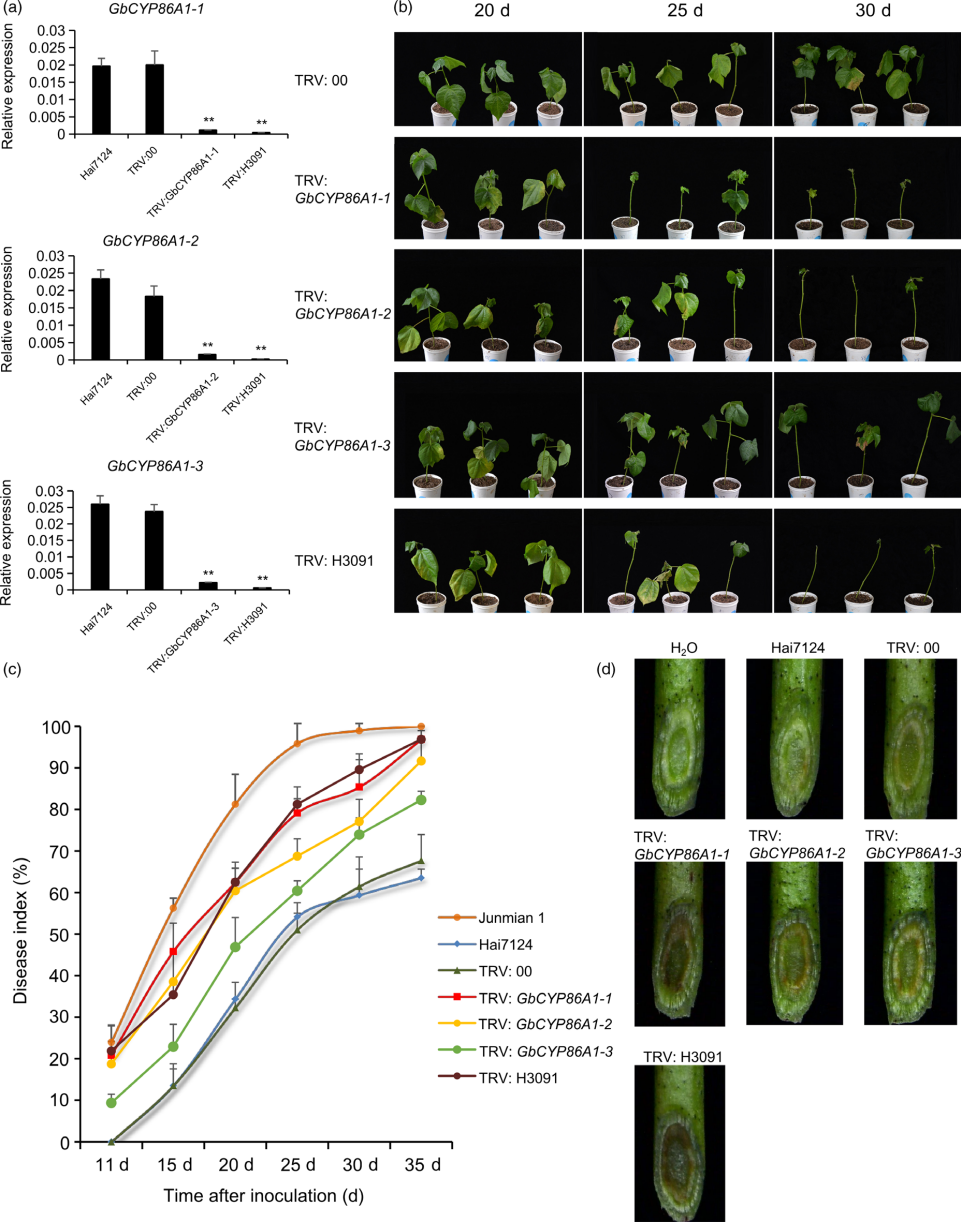
Silencing of *GbCYP86A1‐1*,* GbCYP86A1‐2*,* GbCYP86A1‐3* and H3091 in the resistant cultivar *G. barbadense* cv. Hai7124 leads to increased susceptibility to *Verticillium dahliae* infection. Gene‐specific DNA fragment for specifically silencing one of the three *GbCYP86A1*s and conserved region of these three genes (named as H3091) were selected as the target, respectively.(a) Verification of *GbCYP86A1‐1*,* GbCYP86A1‐2* and *GbCYP86A1‐3* silencing by qRT–PCR in different VIGS plants. Asterisks indicate statistically significant differences, as determined by Student's *t*‐test (**P < 0.01). (b) Disease symptoms of *GbCYP86A1‐1*,* GbCYP86A1‐2*,* GbCYP86A1‐3* and H3091 silenced cotton plants at 20, 25 and 30 days after *V. dahliae* inoculation. (c)Disease progression curves in *GbCYP86A1‐1*,* GbCYP86A1‐2*,* GbCYP86A1‐3* and H3091 silenced cotton plants after *V. dahliae* inoculation. Each biological repeat contains at least 30 seedlings. Error bars represent the standard deviation of three biological replicates. (d) Vascular discoloration in *GbCYP86A1‐1*,* GbCYP86A1‐2*,* GbCYP86A1‐3* and H3091 silenced cotton plants compared with the controls (Hai7124 and TRV:00) after inoculation with V991 and Hai7124 without inoculation(H_2_O). Photographs were taken by stereoscope (Olympus MVX10, Tokyo, Japan) at 15 dpi.

To elucidate the role of GbCYP86A1s in cotton defence against *V. dahliae*, we used VIGS to specifically silence one of the three *GbCYP86A1* genes (marked as TRV2: *GbCYP86A1‐1*, TRV2: *GbCYP86A1‐2* and TRV2: *GbCYP86A1‐3*) and silenced simultaneously the three genes by selecting their conserved regions (TRV2: H3091) in Hai7124, with TRV: 00 as mock treatment and TRV: *GbCLA1* as positive control. As expected, the cotton leaves showed obvious photobleaching phenotype two weeks after agroinfiltration with TRV: *GbCLA1* (Figure S4a). Further, the cotton seedlings infiltrated with different constructs were sampled for RNA isolation and qRT–PCR analysis. The expression of *GbCYP86A1‐1*,* GbCYP86A1‐2* and *GbCYP86A1‐3* was significantly reduced in the separately silenced plants compared with mock‐treated plants, and the expression of three genes in the cosuppression plants by TRV2: H3091 was also significantly reduced (*P *< 0.01) (Figure 2a). Off‐target silencing of other homologs showed that the three homologs had no obvious influence each other in the each specifically silenced plants(Figure S4b).

With Hai7124 and Junmian 1 plants as resistant and susceptible controls to *V. dahliae*, respectively (Figure S4c), after *V. dahliae* strain V991 inoculation, the cotton seedlings of the silenced plants, particularly those containing TRV2: *GbCYP86A1‐1*, TRV2: *GbCYP86A1‐2* and TRV2: H3091, exhibited more wilting, etiolated and even abscission of leaves than control, especially in *GbCYP86A1‐1*‐ and H3091‐silenced plants (Figure 2b). We used at least 30 plants per treatment to calculate the disease index and found that the disease index of TRV2: 00 control plants was approximately 61.5% at 30 days after inoculation. However, the disease index in the *GbCYP86A1‐1*‐silenced and H3091‐silenced plants reached 85.4% and 89.6%, respectively. In addition, the disease index of *GbCYP86A1‐2*‐silenced plants and *GbCYP86A1‐3*‐silenced plants was 77.1% and 74.0%, respectively (Figure 2c; Table S2). The stereomicroscope was used to visually observe the accumulation of invading *V. dahliae* in vascular tissues; *GbCYP86A1‐1*‐silenced and H3091‐silenced plants were more severely affected than control plants (Figure 2d). These findings suggest that after silencing *GbCYP86A1s*, the disease resistance of the plants was weakened to varying degrees. Knocking down *GbCYP86A1‐1* led to a phenotype that was more susceptible to *V. dahliae* infection compared with other two homologs.

### Chromosome location, phylogenetic analysis and subcellular localization of GbCYP86A1s

**Figure 3:**
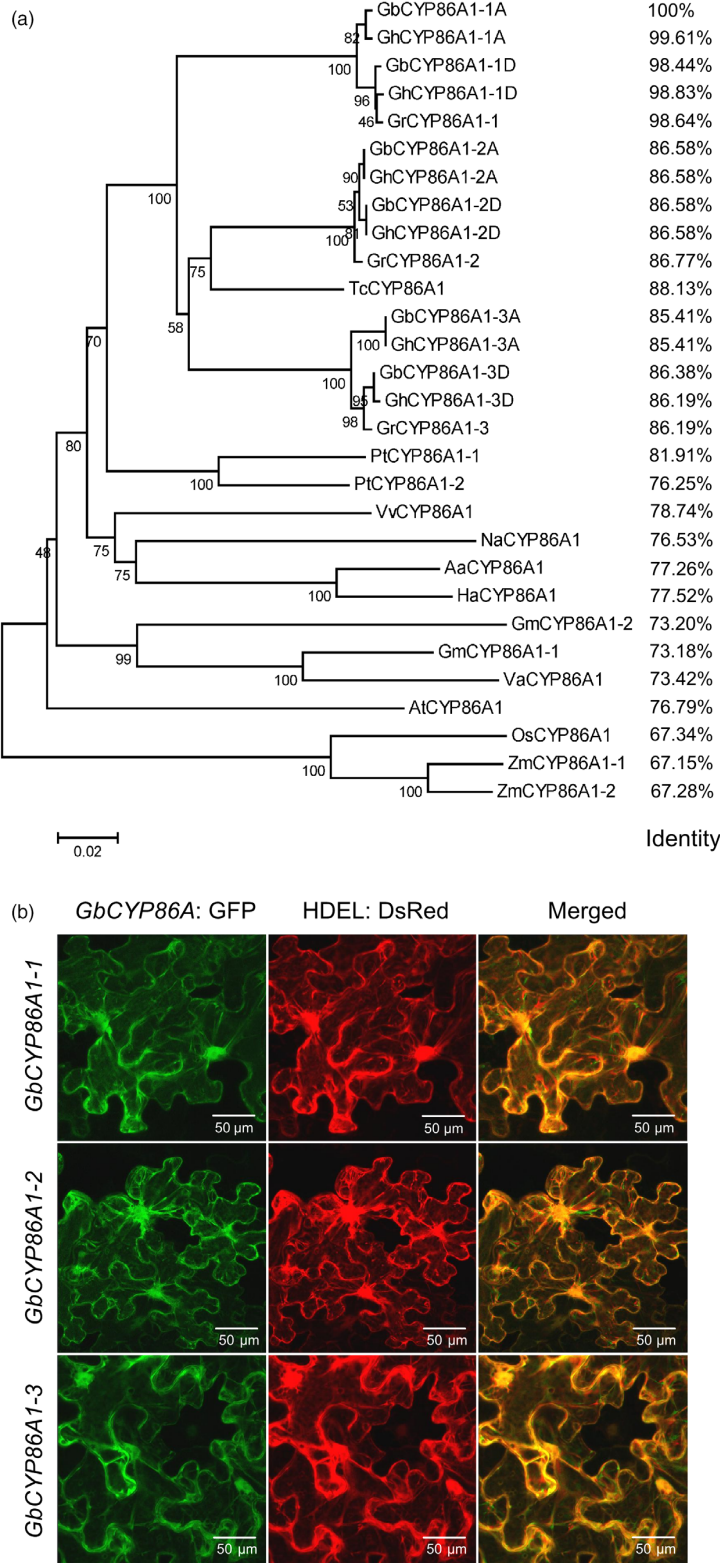
Characterization of *GbCYP86A1* genes. (a) Phylogenetic trees of CYP86A1s from *G. raimondii* (Gr), *G. hirsutum* (Gh), *G. barbadense* (Gb), *Theobroma cacao* (Tc), *Populus trichocarpa* (Pt), *Vitis vinifera* (Vv), *Nicotiana attenuata* (Na), *Artemisia annua* (Aa), *Helianthus annuus* (Ha), *Glycine max* (Gm), *Vigna angularis* (Va), *Arabidopsis thaliana* (At), *Oryza sativa* (Os) and *Zea mays* (Zm) plants. The neighbour‐joining tree was constructed using the MEGA5.1 program ( http://www.megasoftware.net/). Identity value was relative to *GbCYP86A1‐1* and was calculated using software DNAMAN ( http://www.lynnon.com/). (b) Localization of GbCYP86A1s in tobacco epidermal cells by GFP fusion. HDEL: DsRed (red) is an endoplasmic reticulum (ER) marker. The results show that GbCYP86A1s colocalize with the ER marker. Bars = 50 μm.

Chromosome location showed that *CYP86A1s* distributed on different chromosomes with good collinearity in *G. raimondii* and *G. barbadense* (Figure S5). Phylogenetic analysis of GbCYP86A1 orthologs from 12 species showed that CYP86A1s are widely found in different species, and *CYP86A1‐1*,* CYP86A1‐2* and *CYP86A1‐3* can be clearly distinguished into three branches in cotton, with higher homology between *CYP86A1‐2* and *CYP86A1‐3*. Besides *Gossypium*, the *CYP86A1* genes in cotton have the closest homology to cacao and poplar, and more than 75% with *Arabidopsis*, indicating that CYP86A1 may have similar functions in different plants (Figure 3a). Subcellular localization showed that all three GbCYP86A1 proteins were present in ER (Figure 3b), which is consistent with their function in the suberin biosynthesis pathway.

### Functional identification in *V. dahliae* resistance by overexpressing GbCYP86A1s in *Arabidopsis*

**Figure 4:**
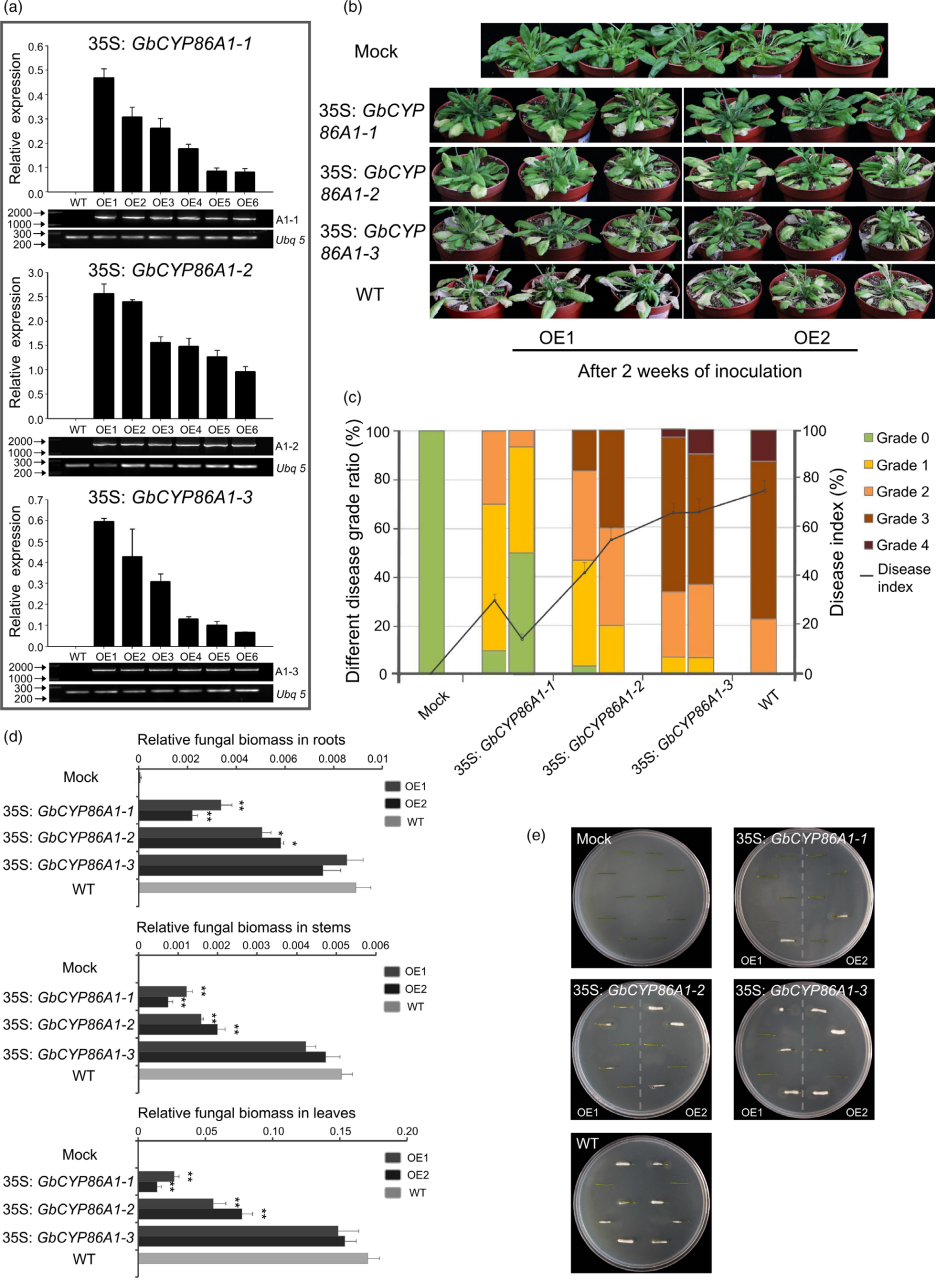
*GbCYP86A1s* overexpression confers resistance to *Verticillium dahliae* infection in *Arabidopsis*. (a) Identification of *Arabidopsis* transgenic lines overexpressing *GbCYP86A1s*. The presence of *GbCYP86A1s* genes was verified by PCR using genomic DNA (lower panel), and *GbCYP86A1* transcript levels in each line were quantified by qRT–PCR using *AtUbq5* (At3g62250) as the internal control (upper panel). WT,* Arabidopsis* Columbia‐0 (Col‐0); OE1 to OE6 represents the different *GbCYP86A1* overexpression lines. Error bars represent the standard deviation of three biological replicates.(b) *GbCYP86A1s* overexpression enhanced resistance to *V. dahliae* infection in *Arabidopsis*. 4‐week‐old *Arabidopsis* plants were inoculated with *V. dahliae* and replanted in soil, with at least 30 plants for each line, and photographed two weeks after inoculation. The mock plants were WT without *V. dahliae* infection. (c) Statistical analysis of disease grade and disease index in transgenic *Arabidopsis* plants and the WT control. The disease grade was classified as five levels as described in Materials and methods. The data were generated from three replicates, with each contains 30 *Arabidopsis* plants. (d) qPCR analysis of fungal biomass in different transgenic and the WT *Arabidopsis* plants. DNAs of roots, stems and leaves were extracted from plants 10 days post‐inoculation by *V. dahliae*. Error bars represent the standard deviation of three biological replicates. Statistical analyses were performed using Student's *t*‐test (*P < 0.05, **P < 0.01). (e) Fungal recovery experiments. Stem sections of *Arabidopsis* transgenic lines and the WT control 7 days post‐inoculation were cut and placed on potato dextrose agar plates and incubated at 25°C. Photographs were taken at 3d after culture.

We generated transgenic *Arabidopsis* lines that heterologously expressed *GbCYP86A1‐1*,* GbCYP86A1‐2* and *GbCYP86A1‐3*, respectively. For each construct, more than ten independent transgenic lines were obtained, and six well‐grown strains from each homozygous T3 line were used for genomic and transcript level identification (Figure 4a). Further, two transgenic lines with high expression were chosen for further analysis.

All the transgenic and WT plants were cultured for four weeks under the same conditions, and no distinct phenotypic differences were observed in growth and development (Figure S6). After V991 inoculation, a more resistant phenotype could be observed in *GbCYP86A1‐1* transgenic line, with less wilting, chlorosis, early senescence and necrosis present. Although the disease resistance of the *GbCYP86A1‐2* transgenic line was also improved compared to WT, it was lower than that in the *GbCYP86A1‐1* transgenic line. In addition, the disease resistance of the *GbCYP86A1‐3* transgenic line was not significantly different to WT plants (Figure 4b). We used at least 30 plants per treatment to calculate the disease index. Two weeks after infection, the disease index of WT plants reached 72.5%; however, the two *GbCYP86A1‐1* transgenic lines were only 30% and 14%, respectively (Figure 4c; Table S3). Fungal biomass analysis confirmed that the degree of fungal invasion in different tissues was altered in the transgenic plants; there was significantly fewer fungal biomass accumulated in the tissues of the *GbCYP86A1‐1* and *GbCYP86A1‐2* transgenic line, especially in *GbCYP86A1‐1* transgenic line. The fungal biomass of the *GbCYP86A1‐3* transgenic line was not significantly different to WT (Figure 4d). A recovery assay to examine the degree of colonization of *V. dahliae* in infected stems also showed that only a small number of colonies were present in the stems of *GbCYP86A1‐1* and *GbCYP86A1‐2* transgenic lines compared to WT and *GbCYP86A1‐3* transgenic lines. The *GbCYP86A1‐1* transgenic line, in particular, showed a significantly enhanced resistance to *V. dahliae* (Figure 4e).

### *GbCYP86A1‐1* contributes to greater suberin accumulation in cell walls and hinders the invasion of *V. dahliae* in *Arabidopsis* roots

**Figure 5:**
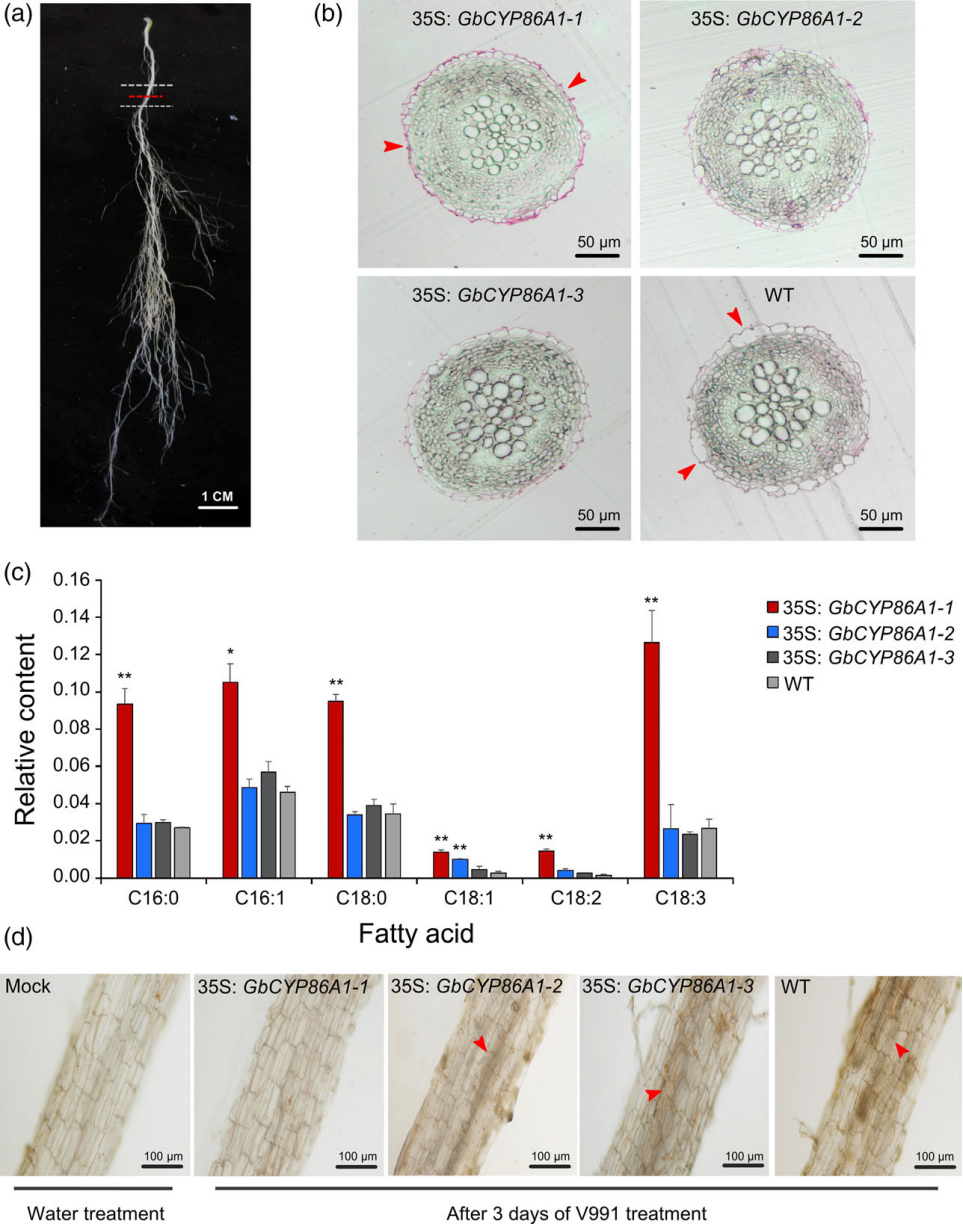
*GbCYP86A1‐1* overexpression promotes lipid production in cell walls and hinders root invasion by *Verticillium dahliae* in *Arabidopsis*. (a) Zone of *Arabidopsis* root used for cross section analysis. The cross section was marked by red dashed line. (b) Bright field microscopic picture of representative cross sections of *Arabidopsis* root stained with the lipophilic dye Sudan 7B. The red‐stained parts represent suberin deposited in the periderm of the roots in secondary developmental stage cell walls. Red arrow marks the periderm cell. Bars = 50 μm. (c) Relative content of fatty acid in the WT and transgenic line roots. Error bars represent the standard deviation of three biological replicates. The asterisks indicate statistically significant differences between the transgenic and WT plants (*P < 0.05, **P < 0.01, Student's *t*‐test). (d) Visualization of *V. dahliae* accumulation in *Arabidopsis* root. Bright field microscopic picture showed representative longitudinal section of the *Arabidopsis* root and was taken 3 days post‐inoculation, Bars = 100 μm. The red arrow marks the invading black mycelium.

Based on these results, we individually selected a transgenic line with the greatest resistance phenotype for each construct, *GbCYP86A1‐1* from OE2 and *GbCYP86A1‐2* and *GbCYP86A1‐3* from OE1, for further analysis. No significant difference in root length, root cell length and root hair length in seedlings of the three *GbCYP86A1*‐overexpressing transgenic *Arabidopsis* was detected (Figure S7).To detect changes in the suberin composition of roots at maturity, we sampled a cross section, 2 cm from the base of the main root (Figure 5a). The sections were stained with the lipophilic dye Sudan 7B and observed, and we found that the cell wall of the outer layer showed greater lipid content in *GbCYP86A1‐1* transgenic line compared with other two transgenic and WT plants, as the red colour was deeper and more obvious (Figure 5b). To assess the metabolic differences between the WT and transgenic plants, fatty acid content was measured. Interestingly, the relative content of C16‐C18 fatty acids in *GbCYP86A1‐1* transgenic line was significantly higher than in the WT (*P *< 0.01). However, the other two transgenic lines did not show any difference, with the exception of higher levels of C18:1 fatty acids in the *GbCYP86A1‐2* transgenic line (Figure 5c). These results indicate that *GbCYP86A1‐1* affects lipid content in peridermal cell walls and fatty acid metabolism in roots, with highly suberized cell walls. In addition, we performed freehand sections and fungal biomass analysis of the root tissue after three days of V991 treatment. Compared with WT and the other two transgenic lines, very little invasion of black mycelium was detected and the relative fungal biomass was also very low in the *GbCYP86A1‐1* transgenic line (Figure 5d; Figure S8). Taken together, the suberized modification of the cell wall in the *GbCYP86A1‐1* transgenic line inhibited the ability of *V. dahliae* to invade and multiply.

### Molecular investigation of the increased disease resistance in the overexpressing *GbCYP86A1‐1* transgenic plants

**Figure 6:**
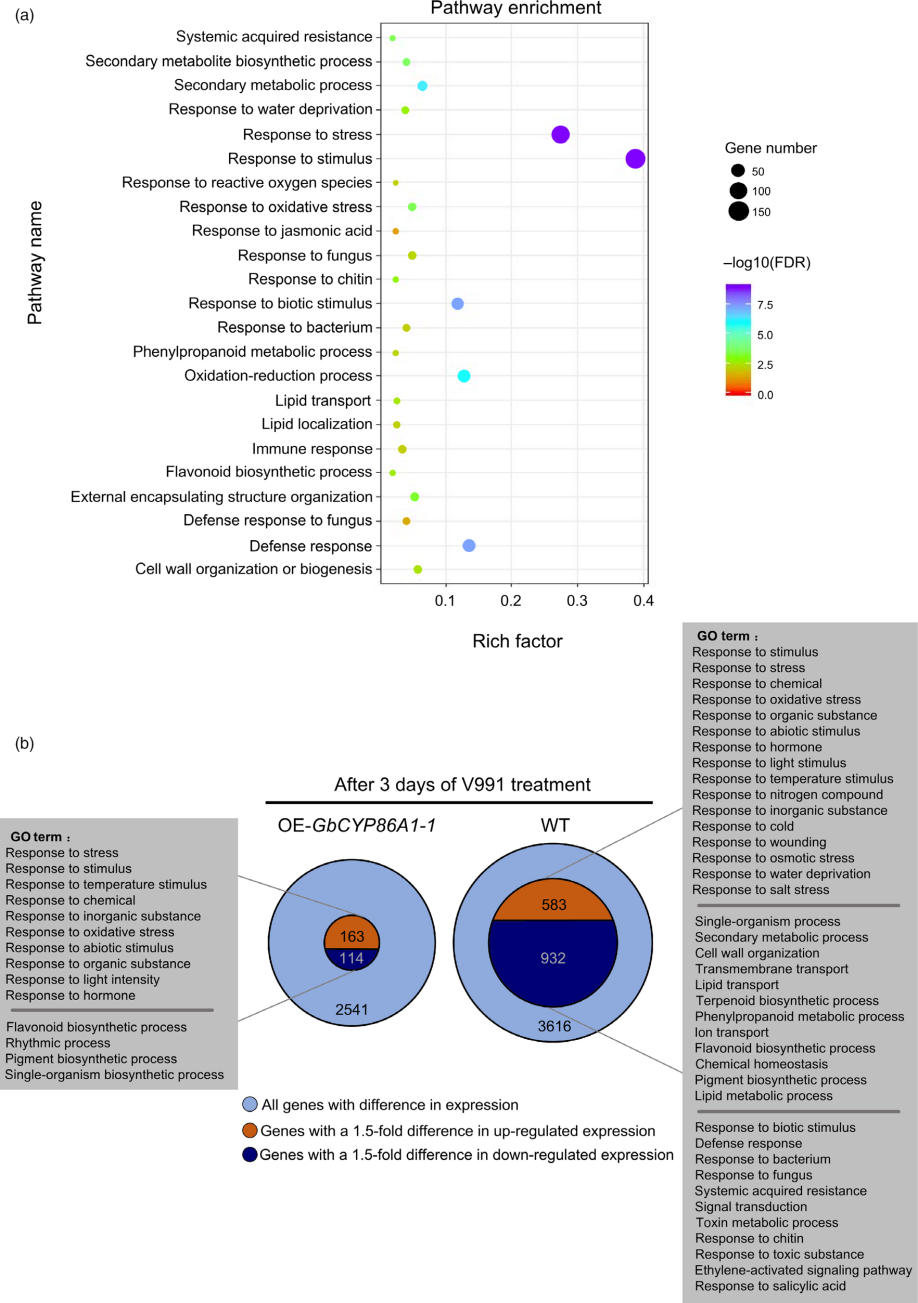
RNA‐seq reveals that *GbCYP86A1‐1* overexpression affects lipid‐related pathways and immunity‐related pathways in root of *Arabidopsis*.(a) GO enrichment analysis of the differentially expressed genes in roots of *GbCYP86A1‐1* transgenic line and the WT control. The RNAs isolated from roots of three individual plants were used for RNA‐seq. *P* value of 0.05 adjusted by false discovery rate (FDR). Rich factor: Percentage of enriched genes comparing with background in corresponding GO term. (b) The number of differentially expressed genes between *GbCYP86A1‐1* transgenic *Arabidopsis* plants and WT after challenged with *Verticillium dahliae* for 3 days. GO terms were separately shown by enrichment analysis. *P* value of 0.05 adjusted by false discovery rate (FDR).

To obtain insights into the molecular basis for the increased resistance of the *GbCYP86A1‐1* transgenic line upon V991 infection, we sampled the roots of the *GbCYP86A1‐1* transgenic plants treated with water and V991 for three days, with WT as a control. Comparative transcriptome analysis between transgenic and WT plants under water treatment showed a total of 481 differential expression genes (q < 0.05 and a fold change > 1.5) (Data S3). With 57 unknown proteins or pseudogenes removed, 222 genes were found to be up‐regulated and 202 genes down‐regulated. Gene ontology (GO) analysis showed that a subset of the genes were further enriched (Figure 6a; Table S4), including those involved in response to stress (FDR = 5.00E‐15), response to stimulus (FDR = 5.30E‐13), response to biotic stimulus (FDR = 4.10E‐08), defence response (FDR = 4.10E‐08), secondary metabolic process (FDR = 5.10E‐07), systemic acquired resistance (FDR = 2.90E‐04), external encapsulating structure (FDR = 6.40E‐04), response to chitin (FDR = 1.50E‐03) and lipid transport (FDR = 2.70E‐03). These overrepresented genes indicate that structural resistance and disease‐resistant immune pathways were initiated in roots of the *GbCYP86A1‐1* transgenic line.

Lipid transfer proteins (LTPs) and ATP‐binding cassette (ABC) transporters are important candidate proteins involved in the transmembrane transport of suberin components (Vishwanath *et al*., 2015). In this study, a number of genes encoding LTPs, ABCs and some AAA‐ATPase family proteins related to ATPase activity had higher expression levels in *GbCYP86A1‐1* transgenic line compared to WT. Further, genes involved in phenylpropanoid metabolism or the synthesis of suberin were also enriched. For example, several P450 superfamily genes, β‐ketoacyl‐CoA synthases (KCS) genes, and genes involved in flavonoid biosynthesis had significantly higher expression in the *GbCYP86A1‐1* transgenic line (Table S5). These results indicate that genes involved in secondary metabolic processes and biosynthesis of suberin were highly induced in *GbCYP86A1‐1* transgenic line.

Changes in cell wall structure or metabolism cause the hydrolysis of pectin and release of oligosaccharides (OGs). OGs released from the plant cell wall as DAMPs can be recognized by receptor RLKs or RLPs, thereby activating downstream immune responses including changes in phytohormones and expression of PRs (Vallarino and Osorio, 2012). Genes encoding pectin lyase‐like superfamily protein and PGIP2, RLKs and RLPs, PRs, ethylene (ET) and salicylic acid (SA) synthesis‐related genes, as well as hormone‐responsive transcription factors, such as ERFs and WRKYs, were significantly up‐regulated in the *GbCYP86A1‐1* transgenic line, while some jasmonate (JA)‐related genes were significantly down‐regulated (Table S5). These integrated results indicate that *GbCYP86A1‐1* affects the immune pathways, activates PR expression and enhances plant disease resistance.

We further compared the differentially expressed genes in the roots of *GbCYP86A1‐1* transgenic and WT plants with inoculated and noninoculated *V. dahliae* treatment. Interestingly, the number of differential genes showed significant differences. In detail, 2,541 differential genes were found in the *GbCYP86A1‐1* transgenic line, 277 of which had a 1.5‐fold change (Data S4). These differential genes were mainly involved in biological processes related to the response to a variety of abiotic factors, as well as growth or metabolism, such as response to temperature stimulus, response to chemical and flavonoid biosynthetic process. In contrast, 3,616 differential genes were found in WT, and 1,515 of these had a 1.5‐fold change (Data S5). In addition to the response to abiotic factors, growth or metabolism, genes mainly involved in disease‐related biological processes were enriched, including those involved in the response to biotic stimuli, defence response, response to fungus, ethylene‐activated signalling pathway, and response to salicylic acid (Figure 6b; Tables S6 and S7). WT roots were attacked by *V. dahliae* and severely damaged internally. In response, more genes were activated, including a large number of genes related to growth or metabolism and resistance genes, in order to prevent further propagation of *V. dahliae*. This might lead to a trade‐off between growth and resistance in plants and show the susceptible phenotype in the WT.

To confirm the RNA‐seq expression data and its reliability, a total of 30 transcripts of secondary metabolism‐related genes, RLKs and RLPs, phytohormone‐related transcription factors and PRs, were selected for qRT–PCR analysis. The qPCR results were highly correlated with RNA‐seq analysis, with the fitting degree above 85% (Figures S9 and S10).

### *GbCYP86A1‐1* knockdown affects the expression of PRs in the roots of *G. barbadense* cv Hai7124

**Figure 7:**
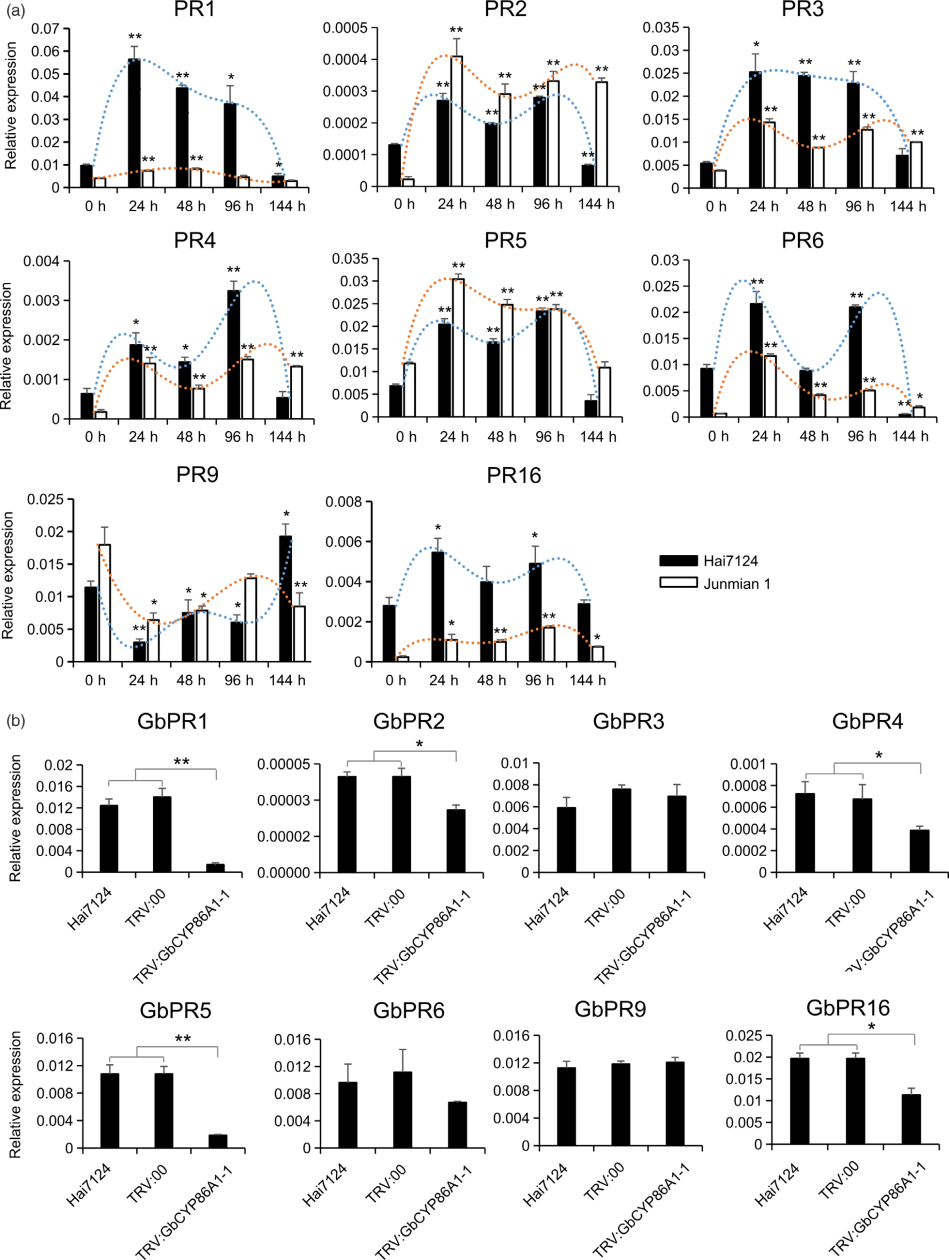
Pathogenesis‐related (PR) gene expression assays.(a) The relative expression of *PR* genes in resistant cultivar Hai7124 and susceptible cultivar Junmian 1 roots after inoculation with *Verticillium dahliae* strain V991. The statistical analyses were performed by comparing expression levels at different time points after *V. dahliae* infection to 0 h without inoculation. (b) The effects of *GbCYP86A1‐1* silencing on PR genes expression in Hai7124. Error bars show the standard deviation of three biological replicates. Statistical analyses were performed using Student's *t*‐test (*P < 0.05, **P < 0.01).

Transcriptome analysis showed that PRs were highly expressed in *GbCYP86A1‐1* transgenic *Arabidopsis*. We analysed the expression patterns of PRs in Hai7124 and Junmian 1 after treatment with V991. Eight genes, PR1, PR2, PR3, PR4, PR5, PR6, PR9 and PR16, were significantly induced in the two cotton cultivars, suggesting their important roles against *V. dahliae* infection in cotton (Figure 7a). To determine whether silencing *GbCYP86A1‐1* affected downstream defence‐related genes in Hai7124, we further analysed the expression of these PRs in the *GbCYP86A1‐1*‐silenced plants. Compared with that in Hai7124 and TRV: 00 control plants, the expression of *GbPR1*,* GbPR2*,* GbPR4*,* GbPR5*,* GbPR16* was significantly lower in *GbCYP86A1‐1*‐silenced plants (Figure 7b), indicating that down‐regulation of *GbCYP86A1‐1* in cotton affects the expression of PRs.

Overall, overexpressing *GbCYP86A1‐1* not only promotes lipid metabolism and transport in roots, leading to the cell wall suberization, but also activates disease‐resistant immune pathways, which prevents *V. dahliae* infection of roots and improves plant disease resistance.

## Discussion

### *GbCYP86A1‐1* is involved in the accumulation of suberin in the roots

Plant roots are protected by specialized lipid‐derived cell wall modifications, such as suberin. The hydrophobic barrier formed by the deposition of suberin is important in controlling the transport of water and nutrients, as well as limiting the invasion of pathogens and toxic compounds in root tissue (Franke and Schreiber, 2007). In this study, several pieces of evidence support the important role of *GbCYP86A1‐1* in root suberization.

It has been demonstrated in *Arabidopsis* and potato that *CYP86A1* mutation results in the destruction of suberin lamellae in roots (Molina *et al*., 2009; Serra *et al*., 2009). In this study, using Sudan 7B staining methods (Höfer *et al*., 2008), we demonstrated that the cell wall of the periderm cells had obvious accumulation of suberin in *GbCYP86A1‐1* transgenic *Arabidopsis* roots. In addition, the relative content of C16‐C18 long‐chain fatty acids increased significantly in transgenic *Arabidopsis* roots, indicating sufficient precursors for lipid synthesis and metabolism.

RNA‐seq sequencing of *GbCYP86A1‐1* transgenic *Arabidopsis* further showed that *GbCYP86A1‐1* affected the biosynthesis and transport of root lipids and affected many transporter proteins in this process, which eventually led to the suberization of the cell wall. In the past decade, great progress has been made in the study of the biosynthesis of suberin monomers. A number of key enzymes and proteins have been discovered in *Arabidopsis* and potato, and the related composition, structure, distribution and biosynthesis of suberin have been reported (Beisson *et al*., 2012). Biosynthesis of suberin monomers is carried out in the ER and involves the hydroxylation of fatty acids, extension of fatty acid precursors, activation of fatty acids to fatty acyl‐CoA thioesters and reduction of fatty acyl chains to fatty alcohols, various acylations largely involving glycerol, phenolic compound incorporation and polymerization of monomers (Pollard *et al*., 2008; Vishwanath *et al*., 2015). Here, we further confirmed that *GbCYP86A1‐1* is located on the ER. Although comparative transcriptome analysis did not enrich significantly the differential expression genes related to C16 and C18 fatty acid biosynthesis, many genes, such as LTPs and ABCs, which encodes proteins involved in the transmembrane transport of suberin components, were up‐regulated in overexpressing *GbCYP86A1‐1* transgenic line. It has been found that *RCN1*/*OsABCG5* in *Oryza sativa* is involved in the suberization of the hypodermis (Shiono *et al*., 2014). *ABCG1* is also involved in the formation of suberin in the periderm of potato tubers (Landgraf *et al*., 2014). *AtABCG2*,* AtABCG6* and *AtABCG20* are involved in the formation of suberin lamellae in the endodermis of roots and seed coats in *Arabidopsis*, and the suberin structure, composition and properties were changed in roots of the corresponding triple mutant, *abcg2*/*abcg6*/*abcg20* (Yadav *et al*., 2014). Recent studies have shown that the nonspecific lipid transfer protein AtLtpI‐4 is required for the formation of suberin in *Arabidopsis* crown gall tumours, and the AtltpI‐4 loss‐of‐function mutant had a significant decrease in the content of suberin components (C18:0) (Deeken *et al*., 2016). AAA family genes have the same ATPase activity as the ABC transporters, and they are involved in the transport of matter in the cell's inner membrane. These are also likely to be important candidate genes for regulating lipid transport (Yedidi *et al*., 2017). In addition, several highly expressed secondary metabolic process‐related genes have also been found, such as KCS is mainly involved in the extension of suberin precursors (Lee *et al*., 2009). The genes encoding GDSL‐like lipase catalyse in *vitro* transesterification of monoacylglycerol precursors (Yeats *et al*., 2014;). Some cytochrome P450 genes such as *CYP86B1* and flavonoid synthesis‐related genes are significantly expressed in transgenic lines, and this may be related to the complexity of plant secondary metabolism (Nesi *et al*., 2000). Taken together, *GbCYP86A1‐1* is involved in the synthesis of suberin in roots and up‐regulates many genes involved in secondary metabolic processes of suberin.

### Cell wall suberization in roots could enhance plant resistance to pathogen

Suberization of the cell wall creates a strong barrier that can make it difficult for pathogens to penetrate and colonize the roots (Andersen *et al*., 2011; Franke and Schreiber, 2007). In‐depth study of suberin biosynthesis may help develop agricultural crops with broad pathogen resistance. Despite extensive evidence on the requirement of CYP86A1 for suberin synthesis (Höfer *et al*., 2008; Molina *et al*., 2009), and the expression of *CYP86A* genes may be affected by some hormones or environmental factors (Duan and Schuler, 2005). However, there have been no reports on its potential role in pathogen resistance. The present analyses show that there are three *GbCYP86A1* homologs in *G. barbadense*. GbCYP86A1‐1 has more three amino acids than GbCYP86A1‐2 and GbCYP86A1‐3 with some amino acid differences among the three homologs. In addition, the *GbCYP86A1s* are specifically expressed in roots and significantly induced by *V.dahliae* with higher expression in disease‐resistant cultivar Hai7124 than disease‐susceptible Junmian 1, implying the potential relationship between suberin and disease resistance involving GbCYP86A1s. Among them, GbCYP86A1‐1 contributed the most significantly to resistance. Silencing of *GbCYP86A1‐1* in Hai7124 led to increased susceptibility, while *GbCYP86A1‐1* overexpression conferred enhanced tolerance and increased suberin in cell walls. In addition, sectional observation also demonstrated that overexpression of *GbCYP86A1‐1* affected the infection of *V. dahliae* in roots.

Another interesting finding is that there were few differentially expressed genes in the roots of *GbCYP86A1‐1* transgenic plants compared with WT when treated with *V. dahliae*. In addition, few differentially expressed genes were enriched for disease‐related pathways. However, the WT roots showed more susceptibility to invasion and destruction by pathogens, with a large amount of genes expressed differentially in order to maintain normal growth and prevent further spread of pathogens. These results provide compelling evidence that *GbCYP86A1‐1* enhances plant disease resistance by promoting accumulation of root suberin and regulating the trade‐off between growth and resistance in plants.

### Overexpression of *GbCYP86A1‐1* activates the disease‐resistant immune pathways

In addition to increasing the structural resistance of cell wall, plants can also activate intracellular complex disease resistance immune pathways to prevent further propagation of pathogens (Jones and Dangl, 2006). Here, we present a new discovery that the suberization of the cell wall boosts the activation of the immune pathway. Plant cell walls are highly heterogeneous extracellular structures that mainly include pectin, cellulose and hemicellulose (Braidwood *et al*., 2014). This structure will be affected by the increase in lipid polymer content in the lamellae of suberized cell walls (Schreiber, 2010). Pectin, as the most complex polysaccharide, constitutes the middle lamella of the cell wall and is mainly composed of polygalacturonan (PG) (Caffall and Mohnen, 2009). OGs can be released from the cell wall as DAMPs by pectin lyase and PG's partial hydrolysis and further activate plant immune responses (Benedetti *et al*., 2015). In addition, PGIPs can interact with PGs and lead to the accumulation of OGs (Mattei *et al*., 2005). Here, through RNA‐seq analysis, we have found that many pectin lyase‐like superfamily proteins (polygalacturonase activity) and PGIP2 expression are activated in *GbCYP86A1‐1* transgenic *Arabidopsis*, which may be associated with changes in the cell wall and lead to the release of OGs. OGs as signalling molecules can be recognized by RLKs or RLPs in the membrane system to activate pathogen‐associated immune responses of plants. A typical example is plant wall‐associated kinase 1 (WAK1) as a receptor for OGs, which rapidly activates the immune system of plants (Gramegna *et al*., 2016). In this study, the expression levels of many RLKs and RLPs were significantly higher in *GbCYP86A1‐1* transgenic *Arabidopsis*, such as *WAK1*,* RLK1* and *RLP38*, compared to WT.

The activation of the immune pathways is mainly reflected by the response of disease‐resistant phytohormones and the expression of PRs (Koornneef and Pieterse, 2008). Compared to the WT, the expression of some genes associated with ET and SA was up‐regulated in *GbCYP86A1‐1* transgenic plants, while JA‐related gene expression was down‐regulated. Among them, the expression of ERFs and WRKYs were significantly up‐regulated. ERFs are not only involved in ethylene‐mediated disease resistance, but also bind to the PR promoter element GCC box and regulate PR expression, so they are also known as pathogenesis‐related transcription factors (Büttner and Singh, 1997; Gutterson and Reuber, 2004). In addition, WRKYs have been recognized as key transcription factors for SA responses and they promote the expression of downstream PRs (Verk *et al*., 2008). PRs can be induced by biotic stresses and in many cases are the marker genes responding to the defence‐associated phytohormones such as ET, SA, JA (Pieterse *et al*., 2009). For example, PR1 (CAP) superfamily proteins are cysteine‐rich secretory proteins that have been frequently used as marker genes for systemic acquired resistance in plants (Lee *et al*., 2015). The PR2 family encodes β‐1,3‐glucanase, a PR2 that may play an important role in *Puccinia triticina* defence response (Mauch *et al*., 1988). The PR5 family encodes permatins, osmotins, zeamatins and thaumatin‐like proteins, and PR5 is the marker gene of the ET signalling pathway in *Brachypodium distachyon* (Kouzai *et al*., 2016). Transgenic *Arabidopsis* expressing PR5 of *Ocimum basilicum* confers tolerance to fungal pathogens (Misra *et al*., 2016). In this study, we found many highly expressed PRs in *GbCYP86A1‐1* transgenic *Arabidopsis*. In addition, we also detected significantly down‐regulated PR expression in *GbCYP86A1‐1*‐silenced plants. Taken together, *GbCYP86A1‐1* is involved in activating downstream immune pathways to enhance resistance to *V. dahliae*.

**Figure 8:**
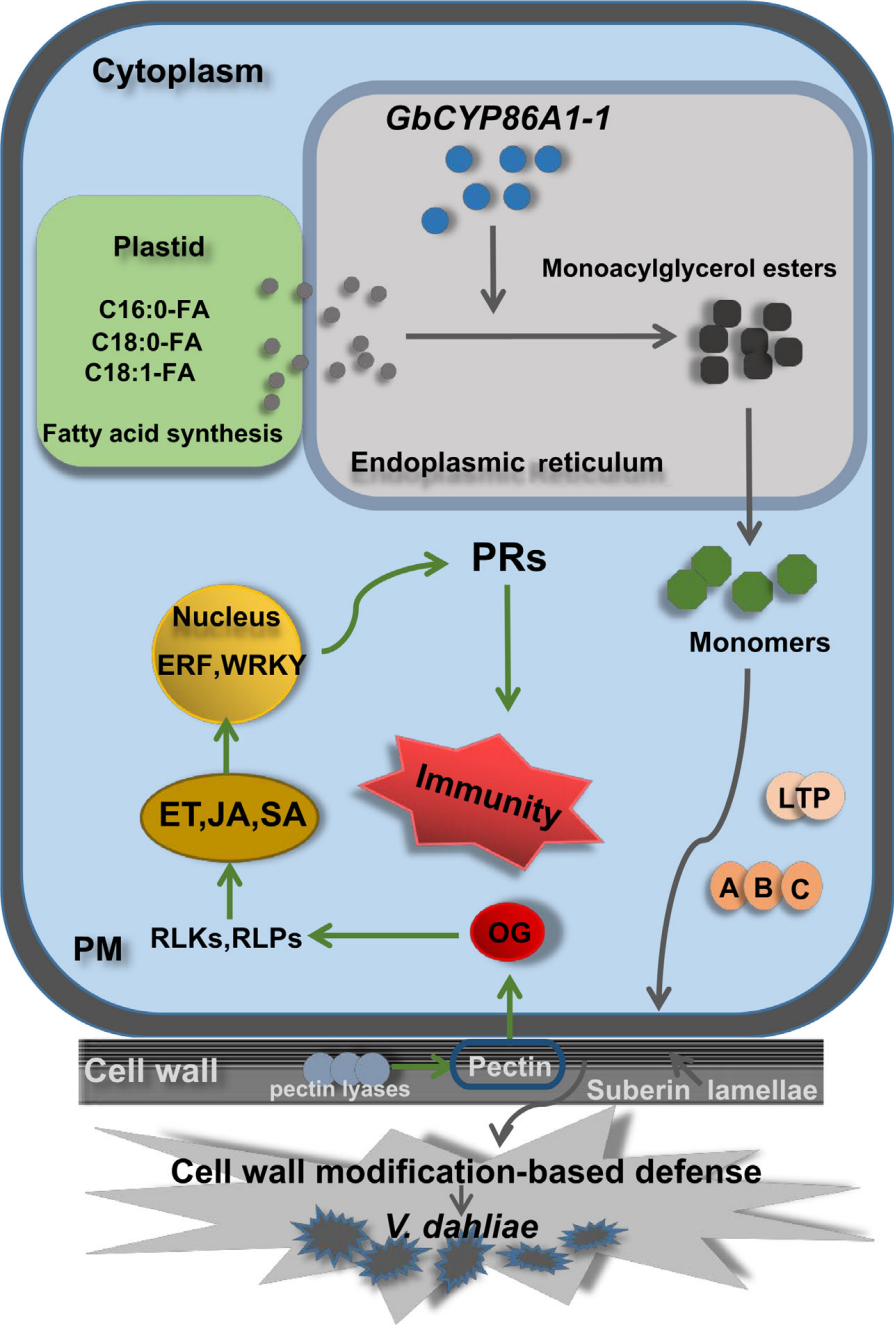
Model of *GbCYP86A1‐1* function in *Verticillium dahliae* –host interaction. The cell wall of plant root epidermis is the first barrier against pathogen attack, and expression of GbCYP86A1‐1 will accelerate suberization in cell walls and induce structural resistance. Fatty acids can be polymerized into the suberin monomers in the endoplasmic reticulum (ER), which then transported to the cell wall. LTPs and ABCs are involved in transport of suberin monomers across the plasma membrane (PM). Suberin accumulating in the cell walls plays a role in defence against the invasion of *V. dahliae*. Additionally, the changed lipids or the cell wall modification may activate intracellular immune responses. Pectate lyase is involved in the hydrolysis of pectin in the cell wall and the release of OGs, which may serve as signalling molecules and activate the immune system. Receptor‐like protein kinases (RLKs) and receptor‐like proteins (RLPs) located in the cell wall or plasma membrane are involved in the recognition and transmission of signals. Production of defence‐related phytohormones, such as ethylene (ET), salicylic acid (SA), jasmonate (JA), and activation of transcription factors (ERF, WRKY) lead to up‐regulation of pathogenesis‐related (PRs) genes and prevent the pathogen propagation.

Based on these, we propose a model of *GbCYP86A1‐1* involvement in plant resistance against *V. dahliae* (Figure 8). GbCYP86A1‐1 proteins were localized to the ER and are likely involved in the suberin biosynthesis. Overexpression of *GbCYP86A1‐1* led to the synthesis of more fatty acids in the plastid, which were activated into fatty acyl‐CoAs in the ER. Fatty acyl‐CoA is oxidized by *GbCYP86A1‐1* to ω‐hydroxy fatty acids and α,ω‐dicarboxylic acids, and this promotes the synthesis of suberin, which is then modified by series of suberin biosynthetic enzymes to form monoacylglycerol esters (Yang *et al*., 2012;). The suberin monomers cross the plasma membrane under the effect of the transport proteins and accumulate in the cell wall. Pectin is the main component of cell wall. Due to cell wall suberization, pectin may be hydrolysed by pectin lyase and release small molecules of OGs. OGs can be detected by members of the receptor protein kinase family or receptor protein family and are responsible for the constitutive activation of pathogen‐related defence responses in plant cells, including initiation of hormone signal transduction and defence gene expression. ERFs and WRKYs are located in the nucleus, in addition to responding to hormones, they can also regulate the expression of disease‐related proteins, further preventing the spread of pathogens. This study presents the novel discovery that *GbCYP86A1‐1* plays important roles in cell wall modification and activation of immune pathways. We are developing the stable *GbCYP86A1‐1* overexpressing and RNAi transgenic upland cotton lines, which will help to thoroughly verify the function of *GbCYP86A1‐1* in cotton. Hopefully, combining *GbCYP86A1‐1* overexpressing cotton lines with traditional breeding techniques will effectively improve the durable disease resistance in cotton.

## Experimental procedures

### Plant materials and treatments

The expression of CYP86s was analysed in *G. barbadense* cv. Hai7124 and *G. hirsutum* cv. Junmian 1 following different treatments. Seedlings were grown in the same controlled environment chamber under the following conditions: 16‐h light/8‐h dark cycle at 28°C for 2 weeks. Wild‐type Arabidopsis (Col‐0) and transgenic *Arabidopsis* were grown in a controlled environmental chamber maintained at 23/21°C (day/night) conditions.

Seedlings of Hai7124 and Junmian 1, which showed resistance and susceptibility to *V. dahliae*, respectively, were inoculated with the fungal pathogen (V991) using the dip‐inoculation method (Wang *et al*., 2011). V991, a highly aggressive and defoliating strain of *V. dahliae* obtained from our lab, was cultured on potato dextrose agar medium (PAD) at 24°C for 4–5 days and then transferred to Czapek's medium for incubation at 25°C for 5 days (Gao *et al*., 2013). Subsequently, we used deionized water to adjust the concentration to 1 × 10^7^ conidia/mL for inoculation of the seedlings. Seedling roots were harvested at 0, 24, 48, 96 and 144 h after V991 treatment for use.

### RNA isolation and expression pattern analysis

Total RNA was isolated from roots using the CTAB‐acidic phenolic method, and the RNA samples were reverse‐transcribed into cDNA using the HiScript Q RT SuperMix for qPCR (+gDNA wiper) (Vazyme). Gene‐specific primers for qRT–PCR analysis were designed using Beacon Designer 7.0. Cotton histone 3 (AF024716) and *Arabidopsis* Ubq5 (At3g62250) were individually used as reference genes. All primer information is shown in Table S8. Real‐time PCRs were performed on a Roche 480 PCR System using AceQ SYBR Green Master(Vazyme).

*G. hirsutum* acc. TM‐1 high‐throughput RNA‐sequencing data from our laboratory (Zhang *et al*., 2015) were used to analyse the expression patterns of GhCYP86s in different tissues. The log_2_ (FPKM) formula was used to calculate their expression. A heat map was generated with Multi Experiment Viewer v.4.9 ( http://en.bio-soft.net/chip/MeV.html).

### Cloning of the CYP86A1s in Hai7124

Gene‐specific primers were designed using Primer 5.0 software to amplify the CYP86A1s with complete ORFs (Table S8). High‐fidelity ExTaq DNA Polymerase (TaKaRa) was used in standard PCR reactions. All PCR products were cloned into pMD19‐T Vectors (TaKaRa) and transformed into strains *E.coli* DH5α. At least six clones per gene were randomly selected and sequenced.

### VIGS experiments

TRV2 vectors were used for VIGS analysis. The 3′ UTR region‐specific sequences were selected to construct TRV2: *GbCYP86A1‐1*, TRV2: *GbCYP86A1‐2* and TRV2: *GbCYP86A1‐3* vectors, and the homologous segments were selected to construct the TRV2: H3091 vector. *GbCLA1* (Cloroplastos alterados 1) was used to construct a TRV2: *GbCLA1* vector. These vectors were transformed into *A. tumefaciens* strain GV3101. Subsequently, *A. tum*efaciens was injected into the cotyledons of cotton seedlings as described previously (Wang *et al*., 2014). Two weeks after injection, RNA was extracted from roots for detecting the expression of target genes. At least 30 plants of each VIGS treatment were selected for *V. dahliae* infection assay by root immersion in a spore suspension. The ratio of diseased to healthy leaves was investigated for seven weeks. According to the percentage of diseased leaves, the disease grades were divided as: 0 (no symptoms), 1 (0–25% wilted leaves), 2 (25–50%), 3 (50–75%) and 4 (75–100%) (Gong *et al*., 2018). Plant disease index (DI) was calculated according to the following formula: DI = [(∑disease grades × number of infected plants) / (total checked plants × 4)] × 100% (Wang *et al*., 2004). To analyse invasive growth in cotton, the stems infected with *V. dahliae* were cut and observed using stereoscope (Olympus MVX10).

### Subcellular localization of GbCYP86A1s

The ORFs of *GbCYP86A1‐1*,* GbCYP86A1‐2* and *GbCYP86A1‐3* were fused to GFP in the pBin‐GFP4 expression vector (Liu *et al*., 2014). The three vectors were transiently expressed in *N. benthamiana* leaf cells via the *A. tumefaciens* infiltration method. The binary vectors were transiently co‐expressed in *N. benthamiana* leaves with RFP‐HDEL via agroinfiltration (Waadt and Kudla, 2008). Fluorescence signals were detected using a confocal laser scanning microscope (Zeiss, LSM710) 3 d after infiltration.

### Identification of transgenic GbCYP86A1s *Arabidopsis* plant resistance

The full‐length sequences of the *GbCYP86A1‐1*,* GbCYP86A1‐2* and *GbCYP86A1‐3* were inserted into the pBI121 vector with the 35S promoter. The overexpression vectors were transferred into GV3101 to transform *Arabidopsis* using the floral dip method. Pure lines were screened in a growth chamber. DNA and RNA were extracted from roots of transgenic lines for detection of positive plants.

For *Arabidopsis* inoculations, 4‐week‐old seedling roots were rinsed in water and incubated in *V. dahliae* conidial suspension (1 × 10^7^ conidia/mL) for 90 seconds, and the plants were then replanted in fresh soil. Severity of disease symptoms was recorded using an index ranging from 0 (healthy plant) to 4 (dead plant) for the disease index calculation (Fradin *et al*., 2011).

For biomass quantification in planta, various tissues were collected for DNA extraction after ten days of treatment with V991. The internal transcribed spacer (ITS) region of ribosomal DNA was targeted using the fungus‐specific ITS1‐F primer in combination with the *V. dahliae* ‐specific reverse primer STVe1‐R. Primers for *AtUbq5* were used as endogenous plant controls (Gutierrez *et al*. 2008). Real‐time PCR was carried out on genomic DNA (Santhanam *et al*., 2013).

To analyse the *V. dahliae* infection rate by recovery assay, stem sections 2 cm from the base were surface sterilized in 70% ethanol and rinsed with sterile water after V991 treatment for a week. The stem segments were placed on the PDA supplemented with chloramphenicol (34 mg/L) and cultured for 3 d at 25°C and then photographed (Gong *et al*., 2018).

### Cellular observation of roots section

*Arabidopsis* plants were grown in vermiculite for four weeks, about 2 cm base of the main root were sampled for cellular observation based on methods described previously (Höfer *et al*., 2008). Samples were dehydrated in ethanol and embedded with resin, stem sections (2 μm thickness) were cut using a Reichert Ultracut microtome for further analysis (Shang *et al*., 2015). Sections were stained with Sudan red 7B and microscopically observed (Olympus BX53) as described previously (Höfer *et al*., 2008).

Freehand sections were prepared from the same location in roots after V991 treatment for 3 days. Samples were fixed in the groove of the carrot, sliced with thin blade and microscopically observed (Olympus BX53).

### Lipid analysis

The 2.244 g potassium hydroxide was added to 100 mL methanol solution to prepare methyl esterification reagent. WT and transgenic line roots were ground to powder, and 0.02 g of sample was placed in 1 mL n‐hexane for total lipid extraction while 2 μg of dotriacontane was added as internal standard. The supernatant was transferred and added 0.5 mL methyl esterification reagent for methylation reaction at room temperature for 1 h (Berry, 2010). The mixtures were then centrifuged, and supernatants were transferred to the sample bottles. Gas chromatography (GC) (Thermo Scientific TRACE GC Ultra) was used to identify C16‐C18 different fatty acids and calculate the area of the peak. A standard curve was first drawn using external standards to test the stability of the GC system and qualitatively analyse different fatty acids. Calculation of relative content was carried out by comparing peak area with internal standards. All analyses are presented as means ± standard deviation of three replicates.

### Transcriptome sequencing

*GbCYP86A1‐1* transgenic and WT plants were grown in vermiculite for four weeks; the roots were collected and ground after 3 days of treatment with V991 or water. Total RNA was isolated and the libraries for sequencing were constructed. Sequencing was performed on the Illumina HiSeq2000 platform. After preprocessing the RNA‐seq data with an NGS QC toolkit, the reads were mapped to the *Arabidopsis* genome (TAIR10) using a Hisat2 with default parameters (Kim *et al*., 2015). Finally, the number of reads aligning to the *Arabidopsis* genome was determined using HTSeq (Anders and Huber, 2010) and imported into R statistical software where differential expression analysis was accomplished using the DESeq with a cut‐off of 0.05 q value and a fold change of > 1.5. GO analysis of the differentially expressed genes in the biological process was conducted using the AgriGO software (Du *et al*., 2010). The background was constituted by the whole annotated gene sequence of *Arabidopsis* and the output of enrichment needed FDR < 0.05. All samples contained three biological repeats.

## Competing interests

The authors declared that they had no competing interests.

## Authors’ contributions

WG conceived the original screening and research plans; GW and JX performed most of the experiments; ZG, LL, GZ and QS provided technical assistance; WG and XW designed the experiments and analysed the data; WG, GW and XW conceived the project and wrote the article with contributions of all the authors; WG and GW supervised and complemented the writing.

## Supporting information


**Figure S1** Phylogenetic relationship of CYP86 subfamily genes in *Arabidopsis* and *G. raimondii*.
**Figure S2** Phylogenetic classification and structural analysis of CYP86 genes in *G. raimondii*.
**Figure S3** Amino acid sequence alignment of GrCYP86 subfamily genes.
**Figure S4** Verification of VIGS silencing system and phenotype of cotton seedlings upon *V. dahliae* inoculation.
**Figure S5** Chromosomal distribution of CYP86 genes in *G. raimondii* and *G. barbadense*.
**Figure S6** Phenotype observation of the above‐ground part of transgenic *Arabidopsis* lines.
**Figure S7** Roots morphology of *Arabidopsis* plants overexpressing *GbCYP86A1s* in seedling stage.
**Figure S8** qPCR analysis of fungal biomass in different transgenic and the WT *Arabidopsis* roots after three days of V991 infection.
**Figure S9** Correlation of fold change analyzed by RNA‐seq data with results obtained from qRT‐PCR.
**Figure S10** Expression patterns of DEGs between WT and *GbCYP86A1‐1* transgenic *Arabidopsis* line related to secondary metabolic processes, RLKs or RLPs, phytohormones‐related transcription factors and PRs.

**Table S1** Comparison of homology between CYP86 family genes in *G. raimondii*.
**Table S2** Disease index of the TRV: *GbCYP86A1‐1*, TRV: *GbCYP86A 1‐2*, TRV: *GbCYP86A1‐3* and TRV*:* H3091 after *V.dahliae* inoculation.
**Table S3** Disease index of the transgenic *Arabidopsis* lines overexpressing *GbCYP86A1‐1*,* GbCYP86A1‐2*, *GbCYP86A1‐3* and WT after *V. dahliae* inoculation.
**Table S4** Genes ontology (GO) analysis involved in biological processes from differentially expressed genes by comparing *GbCYP86A1‐1* transgenic line with WT roots.
**Table S5** Differentially expressed genes by comparing *GbCYP86A1‐1* transgenic line with WT roots.
**Table S6** Genes ontology (GO) analysis involved in biological processes from differential genes by comparing inoculated and un‐inoculated *V. dahliae* in *GbCYP86A1‐1* transgenic line.
**Table S7** Genes ontology (GO) analysis involved in biological processes from differential genes by comparing inoculated and un‐inoculated *V. dahliae* in WT.
**Table S8** Information on PCR primers used in this study.

**Data S1** Identification of CYP86 genes in *G. raimondii* and their phylogenetic relationship in *Arabidopsis thaliana*,* G. arboreum*,* G. hirsutum* and *G. barbadense*, respectively.

**Data S2** Expression profiles of CYP86 genes in *G. hirsutum* acc. TM‐1.

**Data S3** Information on 481 differential expression genes (q < 0.05 and a fold change > 1.5) by transcriptome comparative analysis between transgenic plants and WT.

**Data S4** Information on 277 differential expression genes (q < 0.05 and a fold change > 1.5) by transcriptome comparative analysis between inoculated and non‐inoculated *V. dahliae* treatment in *GbCYP86A1‐1* transgenic line.

**Data S5** Information on 1515 differential expression genes (q < 0.05 and a fold change > 1.5) by transcriptome comparative analysis between inoculated and non‐inoculated *V. dahliae* treatment in WT.
