# Supplementary material for: GbCYP86A1‐1 from Gossypium barbadense positively regulates defence against Verticillium dahliae by cell wall modification and activation of immune pathways
Source: Plant Biotechnol J. 2019 Jun 26;18(1):222–38. doi: 10.1111/pbi.13190 (PMC6920168; doi:10.1111/pbi.13190)
Supplement: Supplementary file 1 — Figure S1 Phylogenetic relationship of CYP86 subfamily genes in Arabidopsis and G. raimondii. Figure S2 Phylogenetic classification and structural analysis of CYP86 genes in G. raimondii. Figure S3 Amino acid sequence alignment of GrCYP86 subfamily genes. Figure S4 Verification of VIGS silencing system and phenotype of cotton seedlings upon V. dahliae inoculation. Figure S5 Chromosomal distribution of CYP86 genes in G. raimondii and G. barbadense. Figure S6 Phenotype observation of the above‐ground part of transgenic Arabidopsis lines. Figure S7 Roots morphology of Arabidopsis plants overexpressing GbCYP86A1s in seedling stage. Figure S8 qPCR analysis of fungal biomass in different transgenic and the WT Arabidopsis roots after three days of V991 infection. Figure S9 Correlation of fold change analyzed by RNA‐seq data with results obtained from qRT‐PCR. Figure S10 Expression patterns of DEGs between WT and GbCYP86A1‐1 transgenic Arabidopsis line related to secondary metabolic processes, RLKs or RLPs, phytohormones‐related transcription factors and PRs. [file PBI-18-222-s006.docx]

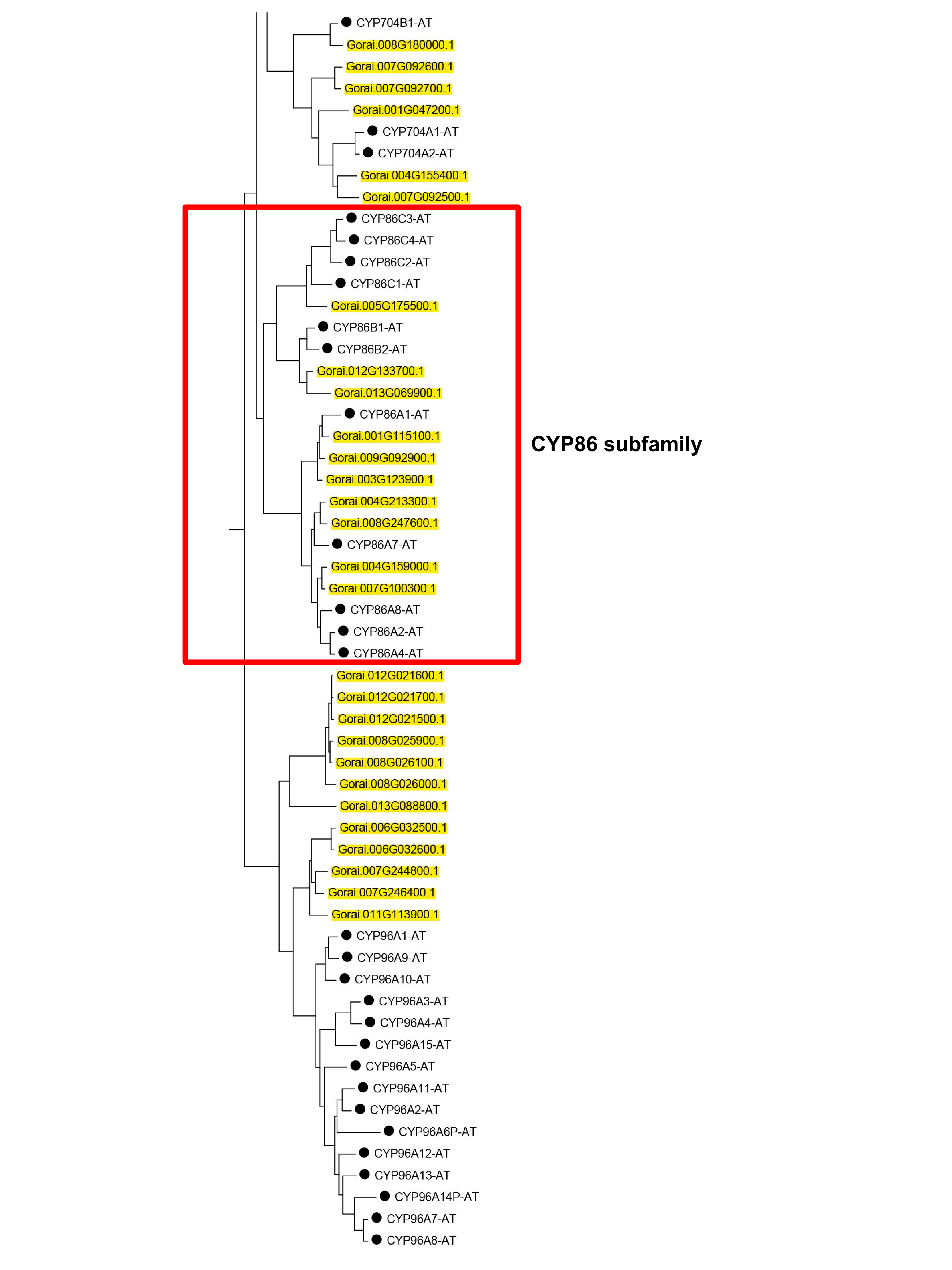


**Figure S1.** Phylogenetic relationship of CYP86 subfamily genes in *Arabidopsis* and *G. raimondii*. Hidden Markov Model profile of the P450 domain (PF00067) was downloaded from the Pfam database (http://pfam.xfam.org/), and then acted as a query to screen P450 proteins using HMMER (V3.0) software **(**<http://hmmer.org/download.html>**)**. The conserved domains of P450 was verified using SMART ([http://smart.embl.de](http://smart.embl.de/)) and INTERPROSCAN software (<http://www.ebi.ac.uk/interpro/>). Amino acid sequences of conserved P450 domain were aligned using ClustalX (version 2.0), and gaps and poorly aligned sections were removed. Phylogenetic tree was generated using the maximum likelihood method under WAG model in MEGA v5.1 (http://www.megasoftware.net/), and the reliability of interior branches was assessed with 1000 bootstrap resamplings. The CYP86 subfamily was circled with the red frame. Based on 11 CYP86 genes in *Arabidopsis*, 10 CYP86 genes were found in *G. raimondii* and clustered together.


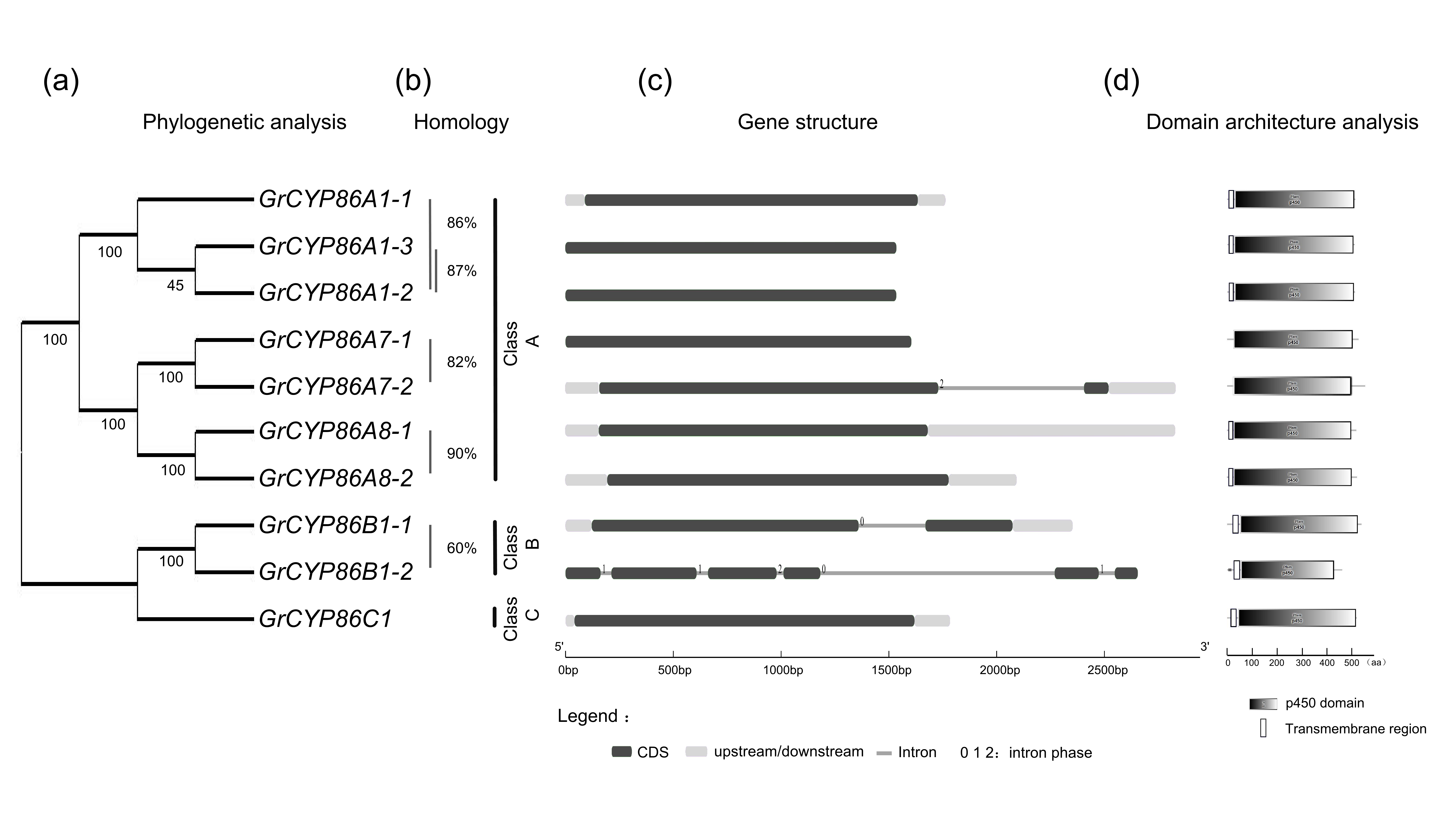


**Figure S2.** Phylogenetic classification and structural analysis of CYP86 genes in *G. raimondii*. (a) Phylogenetic relationship of the ten CYP86 genes in *G. raimondii*. (b) Identity comparison between homologous genes in the CYP86 subfamily. The ten CYP86 genes were clustered into groups A, B and C based on *Arabidopsis* classification. DNAMAN software (http://www.lynnon.com/) was used to compare homology between protein sequences. (c) The gene structures were drawn using the online tool Gene Structure Display Server 2.0 software (<http://gsds.cbi.pku.edu.cn/>). Introns and exons were represented by gray lines and black boxes, respectively, and numbers at the exon-intron joints were intron phases, the gray boxes was the UTR region. The *G. raimondii* genome sequence was available at <http://www.phytozome.net/>. (d) Domain prediction of the ten GrCYP86 proteins. These proteins have the P450 domain, and most contain transmembrane region.


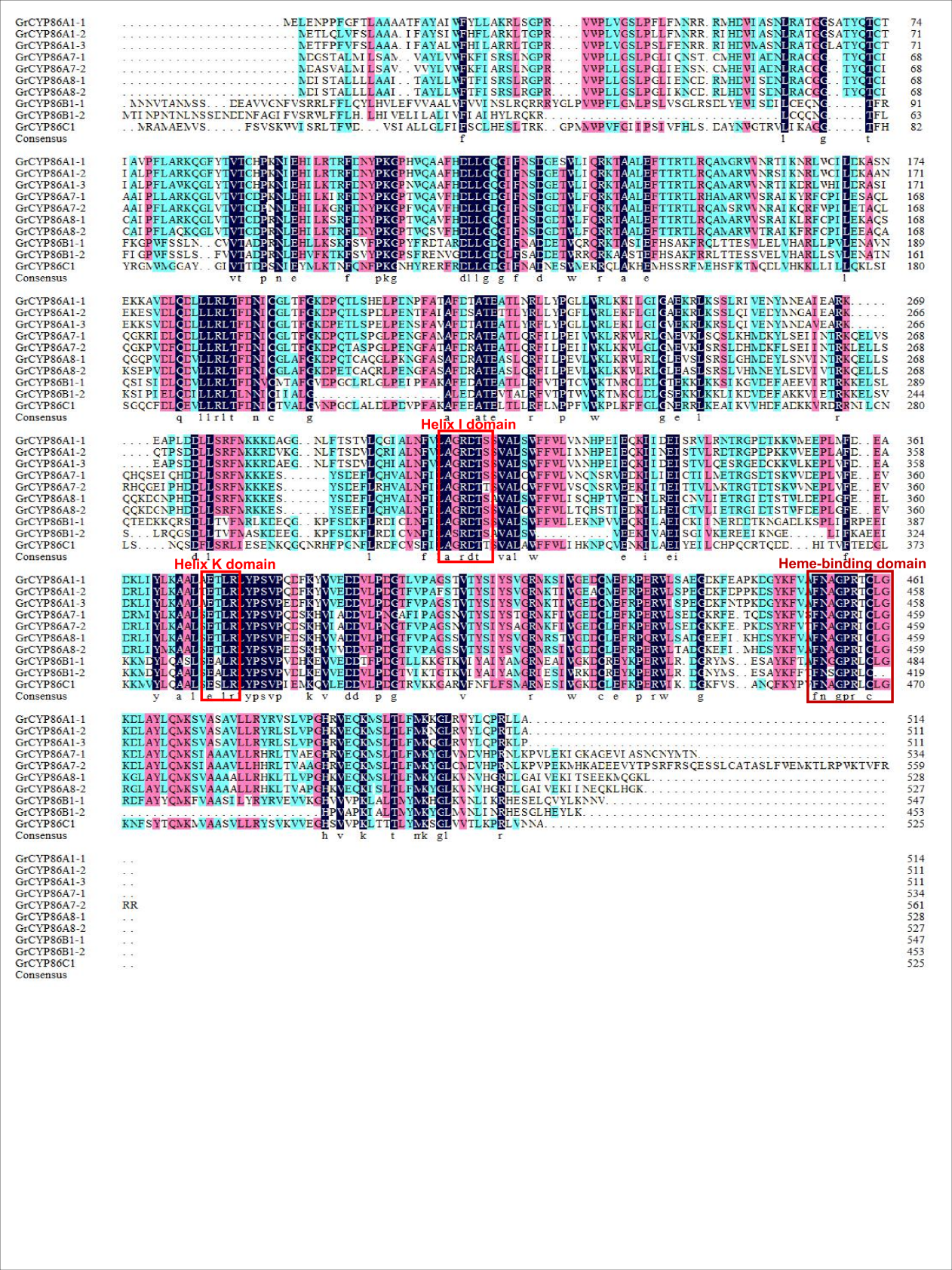


**Figure S3.** Amino acid sequence alignment of GrCYP86 subfamily genes. Amino acid residues highlighted in dark blue and purple represent identical residues in all ten or eight-nine of the aligned protein sequences, respectively. Residues in light blue or white represent residues indicate less similarity in tested sequences, respectively. The GrCYP86 subfamily genes have conserved domains of P450 superfamilies. The Helix I domain, Helix K domain and the Heme-binding domain are marked by the red box.

**
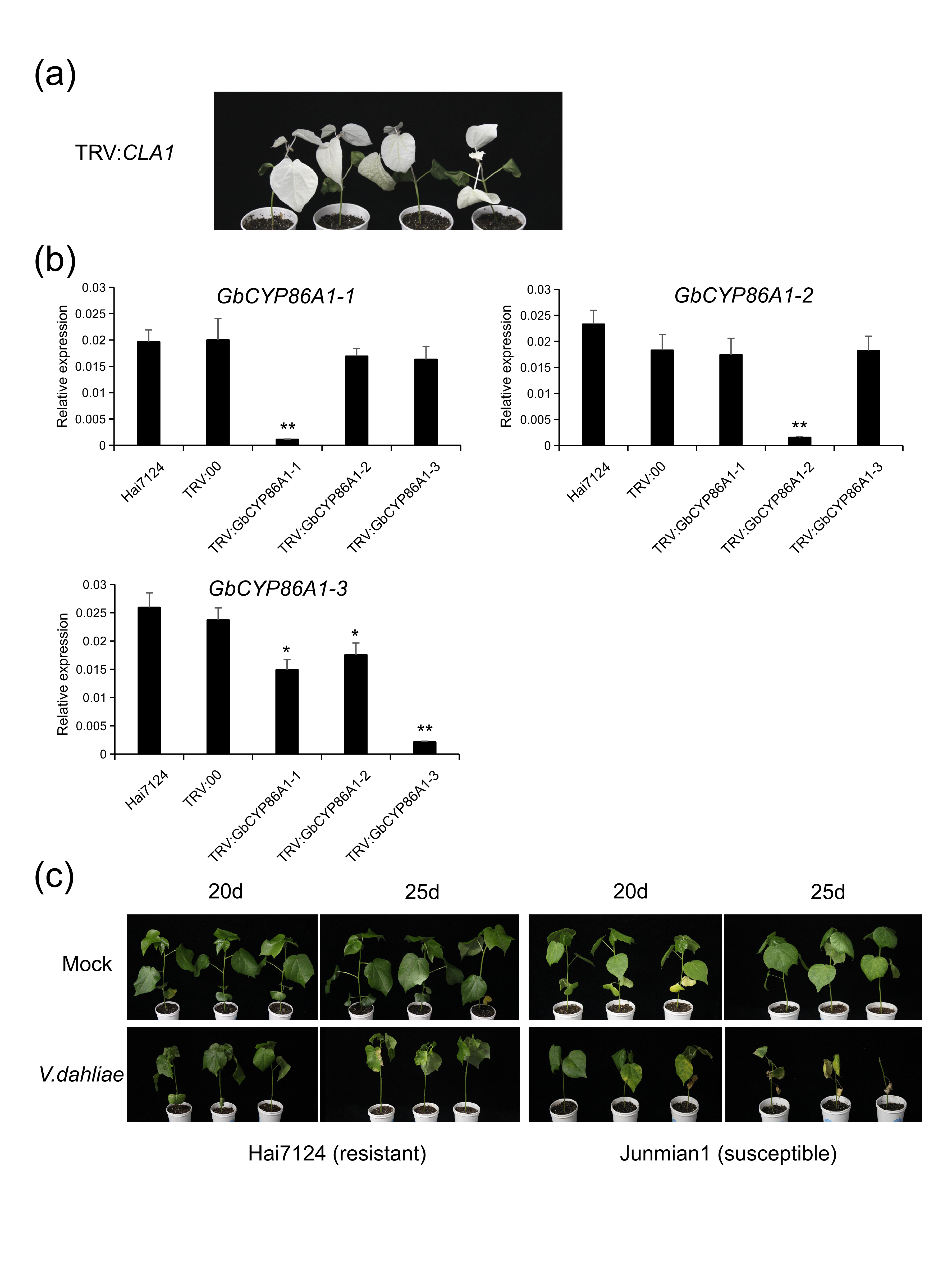
**

**Figure S4.** Verification of VIGS silencing system and phenotype of cotton seedlings upon *V. dahliae* inoculation. (a) Silencing of the endogenous cloroplastos alterados gene (*GbCLA1*) in cotton through tobacco rattle virus (TRV)-mediated virus-induced gene silencing (VIGS). Seven-day-old cotton seedlings (Hai7124) with two fully expanded cotyledons were infiltrated with TRV: *GbCLA1*, and the leaf bleaching phenotype was observed two weeks later. (b) Silencing of the target gene and possible off-target silencing of other GbCYP86A1 homologs was detected, respectively. The error bars were calculated based on three biological replicates using standard deviation. Asterisks indicate statistically significant differences, as determined by Student’s *t*-tests (** P< 0.01, * P< 0.05). (c) Disease symptoms in *G. barbadense* cv. Hai7124 and *G. hirsutum* cv. Junmian 1. The seedlings were grown in the same environment and dip-infected with the liquid containing *V. dahliae* strain V991 spores. We identified the phenotype 20d and 25d after inoculation, confirming that Hai7124 plants were much more resistant than Junmian 1. In Junmian 1, all the true leaves were nearly defoliated 25 days after inoculation.


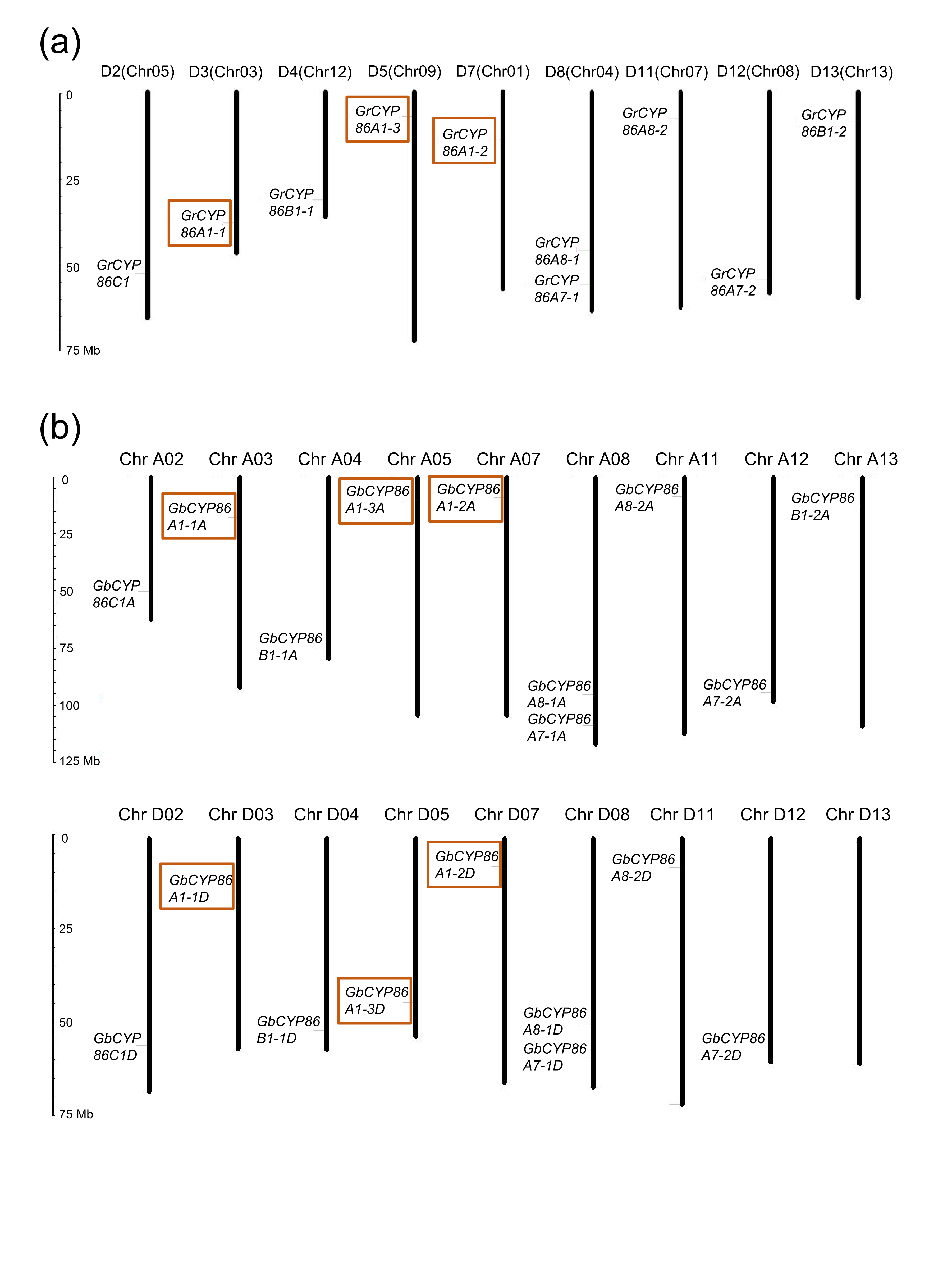


**Figure S5.** Chromosomal distribution of CYP86 genes in *G. raimondii* and *G. barbadense*. (a) Chromosomal distribution of CYP86 genes in *G. raimondii*. By integrating 13 scaffolds of the *G. raimondii* genome (named as Chr01 to Chr13) with the released 26 *G. hirsutum* genome (named as A1 to A13 and D1 to D13), the chromosome numbers are displayed at the top of each bar. The 10 *GrCYP86* genes in *G. raimondii* were marked on the linkage map. (b) The 19 *GbCYP86* genes in *G. barbadense* were marked on the linkage map. The scale represents megabases (Mb). MapInspect software (http://www.softsea.com/review/MapInspect.html) was used to analyze the distribution of CYP86 genes in *Gossypium*. The CYP86A1 genes are circled with red frame. Chromosome location of CYP86 genes showed good collinearity in *G. raimondii* and *G. barbadense*.


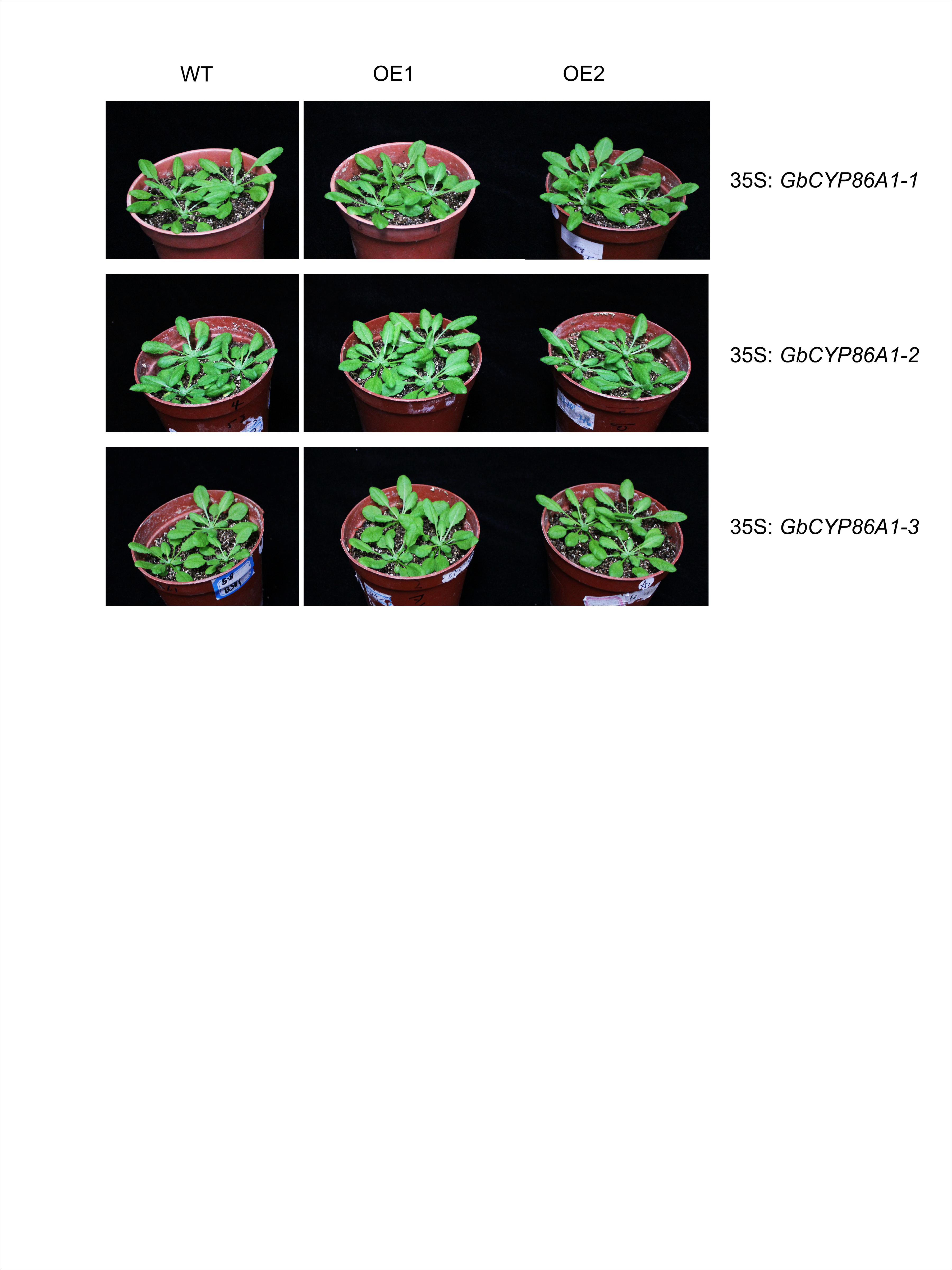


**Figure S6.** Phenotype observation of the above-ground part of transgenic *Arabidopsis* lines. After culturing vertically about 8 days on 1/2 MS solid medium, seedlings were transferred to vermiculite in a growth chamber (21-23°C and approximately 60% relative humidity). Phenotype observations were conducted on 4-week-old plants.


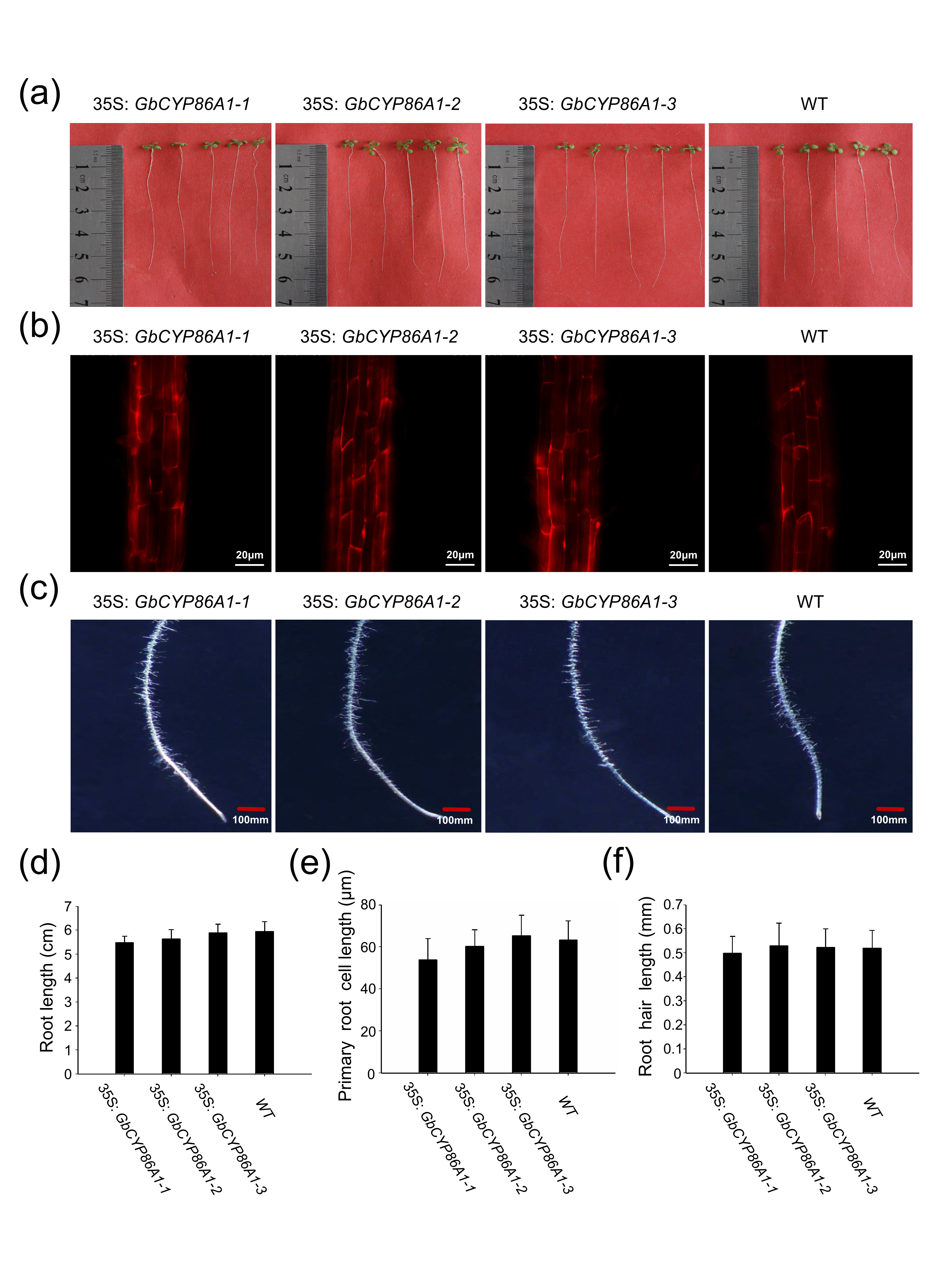


**Figure S7.** Roots morphology of *Arabidopsis* plants overexpressing *GbCYP86A1s* in seedling stage. After disinfection, seeds were sown on 1/2 MS solid medium and then placed vertically into a growth chamber at 23/21°C (day/night). After 8 days, the lengths of the root and root cells from the middle region were measured over 50 biological replicates. (a) Root length of wild-type and *GbCYP86A1s* transgenic *Arabidopsis* lines cultured in culture medium, respectively. (b) Confocal images of elongation zone primary root cells. The root cells were observed after 50 µg/mL propidium iodide (PI) staining for 45s and were observed with a fluorescence microscope (Olympus BX53).These images were taken at similar positions in the elongation zone of each root. Bars=20µm. (c) Photographs of root hairs on wild-type and *GbCYP86A1s* transgenic *Arabidopsis* lines, respectively. The root hair was observed with a stereoscope (Olympus MVX10). Bars=100mm. (d) Histogram of *Arabidopsis* root lengths. Error bars indicate the standard deviation of over 12 seedlings of each transgenic and wild-type *Arabidopsis* plants. (e) Histogram of *Arabidopsis* root cell lengths. The values were averaged over 50 cells from 15 individual seedlings of each transgenic and wild-type *Arabidopsis* plants. (f) Histogram of *Arabidopsis* root hair lengths. The values were averaged over 50 root hairs from 10 individual seedlings of each transgenic and wild-type *Arabidopsis* plants.


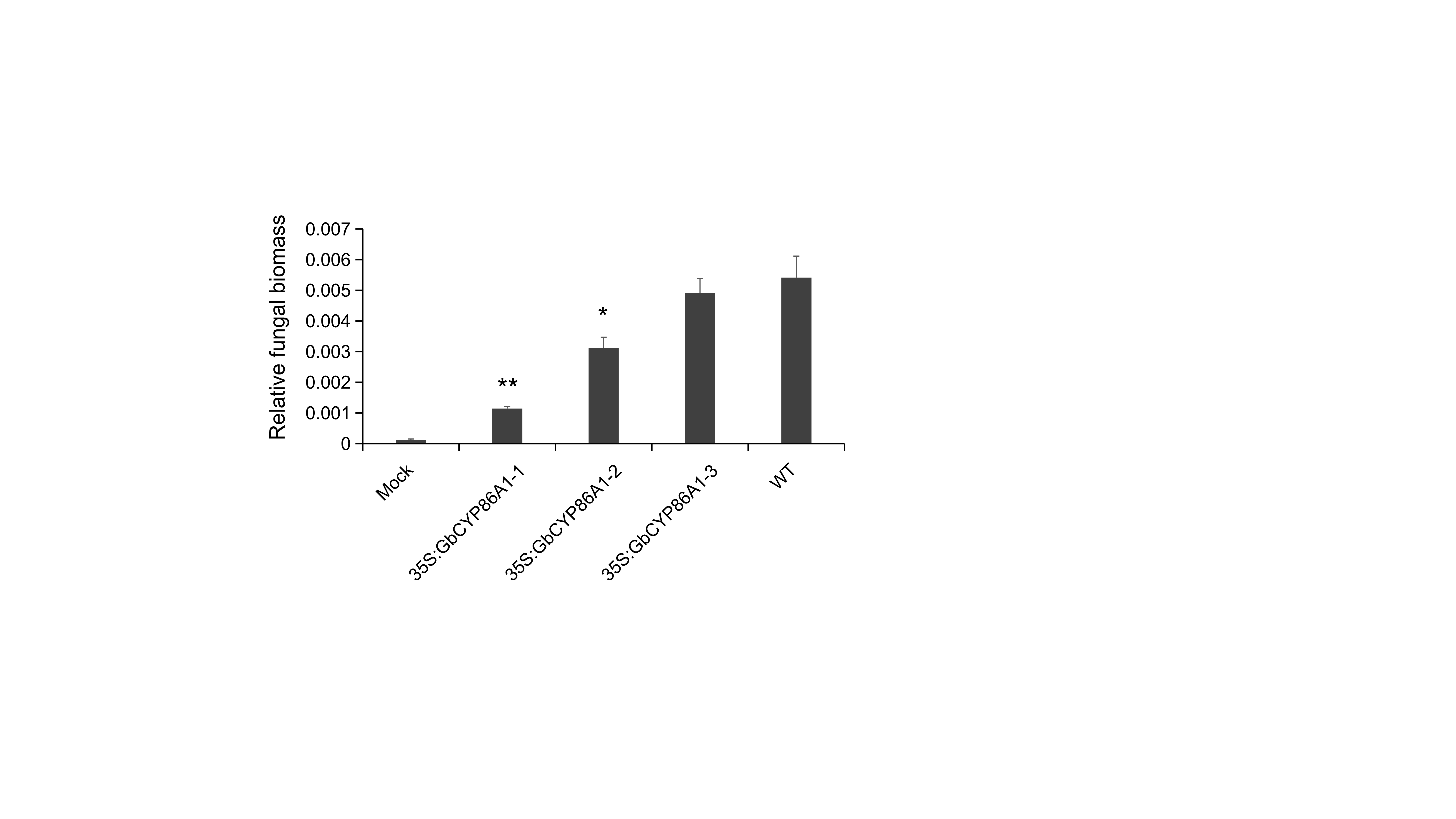


**Figure S8.** qPCR analysis of fungal biomass in different transgenic and the WT *Arabidopsis* roots after three days of V991 infection. The mock plants were WT without *V. dahliae* infection. Error bars showed the standard deviation of three biological replicates. Statistical analyses were performed using Student’s *t*-test to compare differences between transgenic lines and WT (*P < 0.05, **P < 0.01).

**
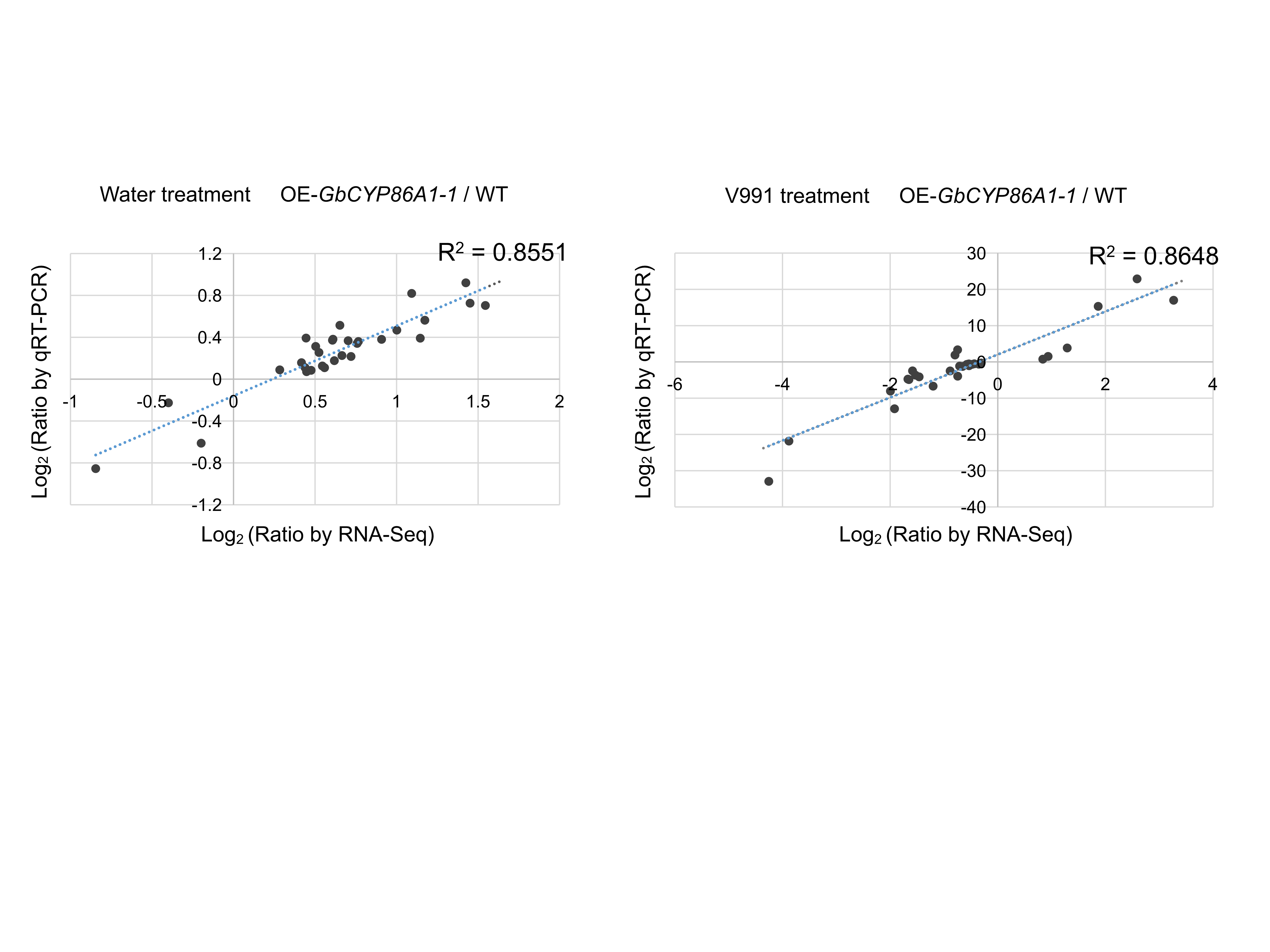
**

**Figure S9.** Correlation of fold change analyzed by RNA-seq data with results obtained from qRT-PCR. Expression data were from 30 genes under all the two conditions (water and V991 treatments) for WT and *GbCYP86A1-1* transgenic *Arabidopsis* line. The RNA-seq log_2_ (expression ratio) values were plotted against the log_2_ (expression ratio) obtained by qRT-PCR, drew standard curves and calculated the *R^2^* value to test the fitness.

**
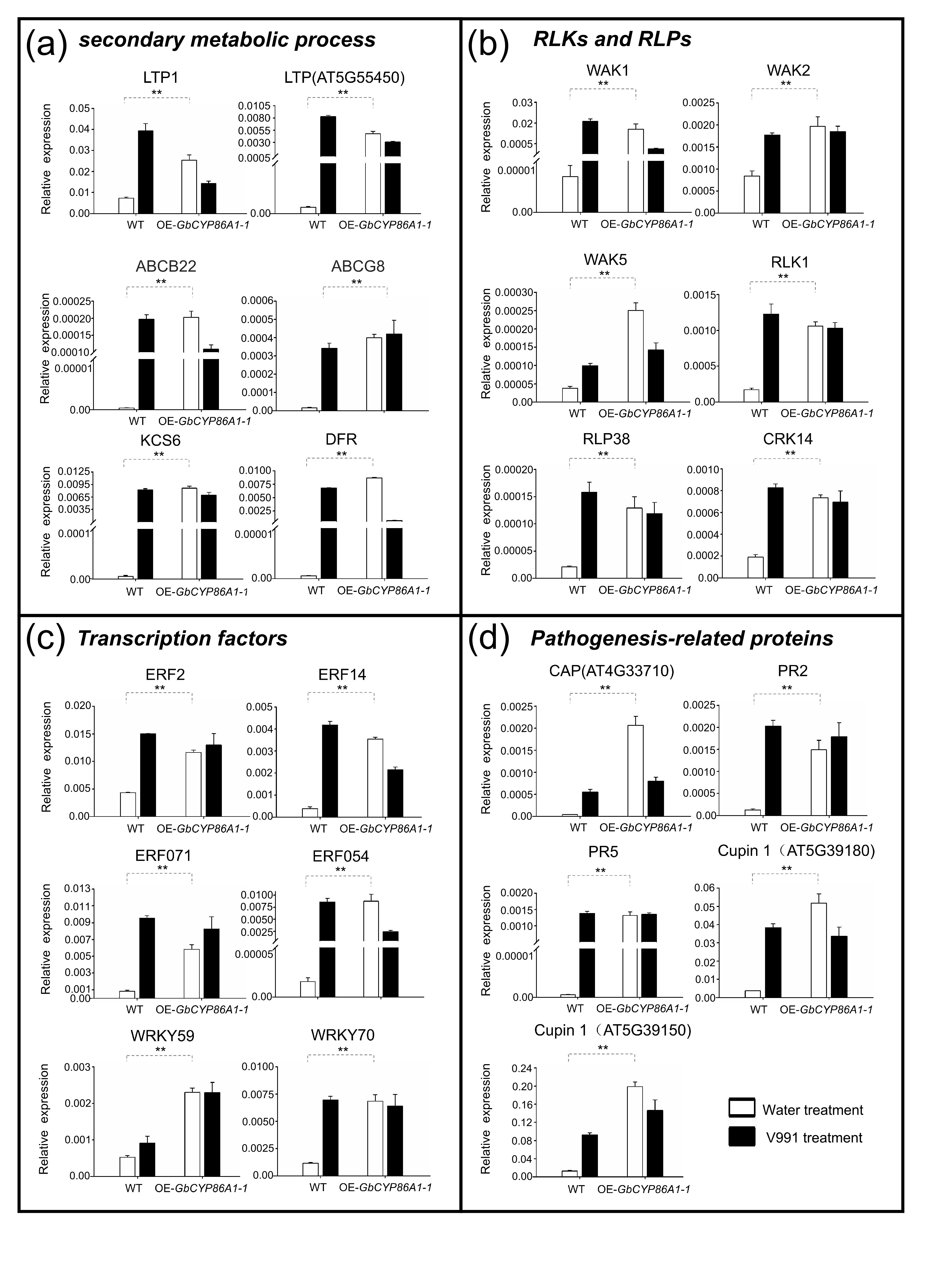
**

**Figure S10.** Expression patterns of DEGs between WT and *GbCYP86A1-1* transgenic *Arabidopsis* line related to secondary metabolic processes, RLKs or RLPs, phytohormones-related transcription factors and PRs. Error bars represent the standard deviation of three independent experiments with three technical replicates for each experiment. Asterisks indicate statistically significant differences, as determined by Student’s *t*-tests (**P< 0.01).
