# Supplementary material for: GbCYP86A1‐1 from Gossypium barbadense positively regulates defence against Verticillium dahliae by cell wall modification and activation of immune pathways
Source: Plant Biotechnol J. 2019 Jun 26;18(1):222–38. doi: 10.1111/pbi.13190 (PMC6920168; doi:10.1111/pbi.13190)
Supplement: Supplementary file 2 — Table S1 Comparison of homology between CYP86 family genes in G. raimondii. Table S2 Disease index of the TRV: GbCYP86A1‐1, TRV: GbCYP86A 1‐2, TRV: GbCYP86A1‐3 and TRV: H3091 after V.dahliae inoculation. Table S3 Disease index of the transgenic Arabidopsis lines overexpressing GbCYP86A1‐1, GbCYP86A1‐2, GbCYP86A1‐3 and WT after V. dahliae inoculation. Table S4 Genes ontology (GO) analysis involved in biological processes from differentially expressed genes by comparing GbCYP86A1‐1 transgenic line with WT roots. Table S5 Differentially expressed genes by comparing GbCYP86A1‐1 transgenic line with WT roots. Table S6 Genes ontology (GO) analysis involved in biological processes from differential genes by comparing inoculated and un‐inoculated V. dahliae in GbCYP86A1‐1 transgenic line. Table S7 Genes ontology (GO) analysis involved in biological processes from differential genes by comparing inoculated and un‐inoculated V. dahliae in WT. Table S8 Information on PCR primers used in this study. [file PBI-18-222-s005.docx]

**Table S1 Comparison of homology between CYP86 family genes in *G. raimondii.***

| Identity (%) | GrCYP86A1-1 | GrCYP86A1-2 | GrCYP86A1-3 | GrCYP86A7-1 | GrCYP86A7-2 | GrCYP86A8-1 | GrCYP86A8-2 | GrCYP86B1-1 | GrCYP86B1-2 | GrCYP86C1 |
| --- | --- | --- | --- | --- | --- | --- | --- | --- | --- | --- |
| GrCYP86A1-1 | 100 |  |  |  |  |  |  |  |  |  |
| GrCYP86A1-2 | 86.38 | 100 |  |  |  |  |  |  |  |  |
| GrCYP86A1-3 | 86.38 | 87.48 | 100 |  |  |  |  |  |  |  |
| GrCYP86A7-1 | 60.92 | 61.44 | 61.62 | 100 |  |  |  |  |  |  |
| GrCYP86A7-2 | 59.51 | 58.95 | 59.65 | 82.38 | 100 |  |  |  |  |  |
| GrCYP86A8-1 | 61.97 | 62.87 | 62.87 | 76.59 | 71.35 | 100 |  |  |  |  |
| GrCYP86A8-2 | 60.59 | 62.06 | 62.62 | 75.28 | 69.93 | 90.15 | 100 |  |  |  |
| GrCYP86B1-1 | 42.01 | 40.04 | 41.29 | 43.64 | 40.07 | 42.86 | 43.47 | 100 |  |  |
| GrCYP86B1-2 | 33.46 | 33.08 | 33.66 | 35.32 | 31.26 | 34.77 | 35.97 | 59.89 | 100 |  |
| GrCYP86C1 | 39.37 | 38.43 | 39.11 | 36.71 | 33.73 | 35.65 | 35.35 | 46.73 | 36.72 | 100 |

DNAMAN software (<http://www.lynnon.com/>) was used to compare homology between protein sequences.

| **Materials** | **Days after *V. dahliae* inoculation and disease index (%)** | | | | | |
| --- | --- | --- | --- | --- | --- | --- |
|  | 11d | 15d | 20d | 25d | 30d | 35d |
| **Junmian 1** | 24.0±4.0 a | 56.3±2.4 a | 81.3±7.2 a | 95.8±5.9 a | 99.0±2.1 a | 100.0±0.0 a |
| **Hai7124** | 0.0±0.0 d | 13.5±4.0 e | 34.4±4.0 d | 54.2±3.4 e | 59.4±6.3 d | 63.5±2.1 e |
| **TRV:00** | 0.0±0.0 d | 13.5±5.2 e | 32.3±2.1 d | 51.0±4.0 e | 61.5±7.1 d | 67.7±6.3 e |
| **TRV:*GbCYP***  ***86A1-1*** | 20.8±3.4 ab | 45.8±6.8 b | 62.5±3.4 b | 79.2±3.4 b | 85.4±8.0 b | 96.9±2.1 b |
| **TRV:*GbCYP***  ***86A1-2*** | 18.8±5.4 b | 38.5±7.1 c | 60.4±5.4 b | 68.8±4.2 c | 77.1±5.4 c | 91.7±4.8 c |
| **TRV:*GbCYP***  ***86A1-3*** | 9.4±2.1 c | 22.9±5.4 d | 46.9±7.1 c | 60.4±2.4 d | 74.0±4.0 c | 82.3±2.1 d |
| **TRV:H3091** | 21.9±6.3 ab | 35.4±2.4 c | 62.5±4.8 b | 81.3±4.2 b | 89.6±2.4 b | 96.9±2.1 b |

**Table S2 Disease index of the TRV: *GbCYP86A1-1,* TRV: *GbCYP86A1-2,* TRV: *GbCYP86A1-3* and TRV*:* H3091 after *V. dahliae* inoculation.**

TRV: *GbCYP86A1-1*, TRV: *GbCYP86A1-2*, TRV: *GbCYP86A1-3* for silencing specifically the corresponding gene, and TRV: H3091 for simultaneously silencing three *GbCYP86A1* homologs. These silenced cotton plants were inoculated by *V. dahliae* with TRV:00 as control. Hai7124 and Junmian 1, which showed resistance and susceptibility to *V. dahliae*, respectively, were also used as controls. The experiments were repeated at least three times and each treatment was applied to more than 30 plants. The disease grade of cotton was investigated and the disease index was calculated, as described in Materials and methods. The standard deviation represent the differences among the three independent biological experiments. Lowercase letters represent the statistical significance (P<0.05, ANOVA).

**Table S3 Disease index of the transgenic *Arabidopsis* lines overexpressing *GbCYP86A1-1*, *GbCYP86A1-2*, *GbCYP86A1-3* and WT after *V. dahliae* inoculation.**

| **Materials** | **Days after *V. dahliae* inoculation** **and disease index (%)** | | |
| --- | --- | --- | --- |
|  | 7d | 14d | 21d |
| Mock | 0 | 0 | 0 |
| 35S: GbCYP86A1-1 OE1 | 12.5±2.5 d | 30.0±2.5 d | 64.2±2.9 c |
| 35S: GbCYP86A1-1 OE2 | 5.0±2.5 e | 14.2±1.4 e | 60.8±2.9 c |
| 35S: GbCYP86A1-2 OE1 | 15.0±2.5 cd | 41.7±3.8 c | 77.5±5.0 b |
| 35S: GbCYP86A1-2 OE2 | 18.3±2.9 c | 55.0±5.0 b | 82.5±5.0 b |
| 35S: GbCYP86A1-3 OE1 | 25.0±2.5 b | 65.8±3.8 a | 94.2±1.4 a |
| 35S: GbCYP86A1-3 OE2 | 27.5±2.5 ab | 66.7±5.2 a | 93.3±2.9 a |
| WT | 30.0±2.5 a | 72.5±4.3 a | 95.0±2.5 a |

Transgenic *Arabidopsis* overexpressing *GbCYP86A1-1*, *GbCYP86A1-2*, *GbCYP86A1-3* lines were respectively inoculated for disease resistance investigation, with wild type *Arabidopsis* inoculated in water (Mock) and *V. dahliae* (WT) as control. The experiments were repeated at least three times and each treatment was applied to more than 30 plants. The disease grade of *Arabidopsis* was investigated and the disease index was calculated, as described in Materials and methods. The standard deviation represent the differences among the three independent biological experiments. Lowercase letters represent the statistical significance (P<0.05, ANOVA).

**Table S4 Genes ontology (GO) analysis involved in biological processes from differentially expressed genes by comparing *GbCYP86A1-1* transgenic line with WT roots.**

| GO_acc. | Term type | Term | Query item | Bg item | P value | FDR |
| --- | --- | --- | --- | --- | --- | --- |
| GO:0006950 | P | response to stress | [128](http://systemsbiology.cau.edu.cn/agriGOv2/termDetail.php?session=589589793.1&GO=GO:0006950) | 3506 | 2.00E-18 | 5.00E-15 |
| GO:0050896 | P | response to stimulus | [180](http://systemsbiology.cau.edu.cn/agriGOv2/termDetail.php?session=589589793.1&GO=GO:0050896) | 6250 | 4.30E-16 | 5.30E-13 |
| GO:0009607 | P | response to biotic stimulus | [55](http://systemsbiology.cau.edu.cn/agriGOv2/termDetail.php?session=589589793.1&GO=GO:0009607) | 1253 | 8.50E-11 | 4.10E-08 |
| GO:0006952 | P | defense response | [63](http://systemsbiology.cau.edu.cn/agriGOv2/termDetail.php?session=589589793.1&GO=GO:0006952) | 1566 | 1.00E-10 | 4.10E-08 |
| GO:0019748 | P | secondary metabolic process | [30](http://systemsbiology.cau.edu.cn/agriGOv2/termDetail.php?session=589589793.1&GO=GO:0019748) | 492 | 2.30E-09 | 5.10E-07 |
| GO:0055114 | P | oxidation-reduction process | [59](http://systemsbiology.cau.edu.cn/agriGOv2/termDetail.php?session=589589793.1&GO=GO:0055114) | 1566 | 4.40E-09 | 9.10E-07 |
| GO:0044550 | P | secondary metabolite biosynthetic process | [19](http://systemsbiology.cau.edu.cn/agriGOv2/termDetail.php?session=589589793.1&GO=GO:0044550) | 311 | 2.00E-06 | 0.0002 |
| GO:0006979 | P | response to oxidative stress | [23](http://systemsbiology.cau.edu.cn/agriGOv2/termDetail.php?session=589589793.1&GO=GO:0006979) | 453 | 3.40E-06 | 0.00028 |
| GO:0009627 | P | systemic acquired resistance | [9](http://systemsbiology.cau.edu.cn/agriGOv2/termDetail.php?session=589589793.1&GO=GO:0009627) | 67 | 3.60E-06 | 0.00029 |
| GO:0045229 | P | external encapsulating structure organization | [25](http://systemsbiology.cau.edu.cn/agriGOv2/termDetail.php?session=589589793.1&GO=GO:0045229) | 551 | 8.40E-06 | 0.00064 |
| GO:0010200 | P | response to chitin | [11](http://systemsbiology.cau.edu.cn/agriGOv2/termDetail.php?session=589589793.1&GO=GO:0010200) | 134 | 2.40E-05 | 0.0015 |
| GO:0009414 | P | response to water deprivation | [18](http://systemsbiology.cau.edu.cn/agriGOv2/termDetail.php?session=589589793.1&GO=GO:0009414) | 347 | 3.00E-05 | 0.0018 |
| GO:0009813 | P | flavonoid biosynthetic process | [9](http://systemsbiology.cau.edu.cn/agriGOv2/termDetail.php?session=589589793.1&GO=GO:0009813) | 93 | 4.00E-05 | 0.0022 |
| GO:0006869 | P | lipid transport | [12](http://systemsbiology.cau.edu.cn/agriGOv2/termDetail.php?session=589589793.1&GO=GO:0006869) | 174 | 5.10E-05 | 0.0027 |
| GO:0071554 | P | cell wall organization or biogenesis | [27](http://systemsbiology.cau.edu.cn/agriGOv2/termDetail.php?session=589589793.1&GO=GO:0071554) | 704 | 6.20E-05 | 0.0032 |
| GO:0009620 | P | response to fungus | [23](http://systemsbiology.cau.edu.cn/agriGOv2/termDetail.php?session=589589793.1&GO=GO:0009620) | 573 | 0.00011 | 0.0054 |
| GO:0009698 | P | phenylpropanoid metabolic process | [11](http://systemsbiology.cau.edu.cn/agriGOv2/termDetail.php?session=589589793.1&GO=GO:0009698) | 164 | 0.00013 | 0.006 |
| GO:0000302 | P | response to reactive oxygen species | [11](http://systemsbiology.cau.edu.cn/agriGOv2/termDetail.php?session=589589793.1&GO=GO:0000302) | 166 | 0.00015 | 0.0064 |
| GO:0010876 | P | lipid localization | [12](http://systemsbiology.cau.edu.cn/agriGOv2/termDetail.php?session=589589793.1&GO=GO:0010876) | 199 | 0.00017 | 0.0072 |
| GO:0006955 | P | immune response | [16](http://systemsbiology.cau.edu.cn/agriGOv2/termDetail.php?session=589589793.1&GO=GO:0006955) | 333 | 0.00019 | 0.0078 |
| GO:0009617 | P | response to bacterium | [19](http://systemsbiology.cau.edu.cn/agriGOv2/termDetail.php?session=589589793.1&GO=GO:0009617) | 441 | 0.00019 | 0.0078 |
| GO:0050832 | P | defense response to fungus | [19](http://systemsbiology.cau.edu.cn/agriGOv2/termDetail.php?session=589589793.1&GO=GO:0050832) | 504 | 0.00091 | 0.03 |
| GO:0009753 | P | response to jasmonic acid | [11](http://systemsbiology.cau.edu.cn/agriGOv2/termDetail.php?session=589589793.1&GO=GO:0009753) | 225 | 0.0016 | 0.049 |
| GO:0002682 | P | regulation of immune system process | 6 | 111 | 0.012 | 0.24 |
| GO:0006629 | P | lipid metabolic process | 26 | 994 | 0.015 | 0.28 |
| GO:0042545 | P | cell wall modification | 7 | 156 | 0.017 | 0.31 |
| GO:0033993 | P | response to lipid | 21 | 772 | 0.018 | 0.34 |

**Table S5 Differentially expressed genes by comparing *GbCYP86A1-1* transgenic line with WT roots.**

| **Gene ID** | **Description** | Fold of change (OE/WT) |
| --- | --- | --- |
| **Activated secondary metabolic process** | | |
| ***Lipid transfer related protein*** | | |
| AT5G55450 | Lipid-transfer protein | 4.29 |
| AT4G12500 | Lipid-transfer protein | 9.64 |
| AT3G22142 | Lipid-transfer protein | 4.75 |
| AT2G38540 | Lipid-transfer protein | 1.81 |
| AT3G22120 | Cell wall-plasma membrane linker protein (CWLP), lipid-transfer protein | 1.74 |
| AT5G48485 | DEFECTIVE IN INDUCED RESISTANCE 1 (DIR1), lipid-transfer protein | 2.5 |
| ***ATPase activity related protein*** | | |
| AT3G28415 | ABC transporter family protein, ABCB22 | 4.85 |
| AT5G52860 | ABC-2 type transporter family protein, ABCG8 | 2.62 |
| AT1G51460 | ABC-2 type transporter family protein, ABCG13 | 1.7 |
| AT3G28380 | P-glycoprotein 17 (PGP17), ABCB17 | 2.44 |
| AT4G25960 | P-glycoprotein 2 (PGP2), ABCB2 | 1.57 |
| AT3G13090 | Multidrug resistance-associated protein 8 (MRP8), ABCC6 | 1.55 |
| AT3G28600 | AAA-ATPase | 3.12 |
| AT3G28510 | AAA-ATPase | 2.14 |
| AT5G40000 | AAA-ATPase | 1.84 |
| AT5G40010 | AAA-ATPase 1 (AATP1) | 1.7 |
| AT3G28580 | AAA-ATPase | 1.69 |
| ***Other secondary metabolic process related protein*** | | |
| AT1G68530 | 3-ketoacyl-CoA synthase 6 (KCS6) | 1.61 |
| AT1G31550 | GDSL - like Lipase | 2.15 |
| AT1G26200 | TRAM, LAG1 and CLN8 (TLC) lipid-sensing domain containing protein | 1.72 |
| AT1G43800 | Plant stearoyl-acyl-carrier-protein desaturase family protein | 1.83 |
| AT5G54060 | Flavonoid 3-o-glucosyltransferase (UF3GT) | 3.53 |
| AT5G42800 | Dihydroflavonol 4-reductase (DFR) | 4.7 |
| AT5G13930 | TRANSPARENT TESTA 4 (TT4) | 3.07 |
| AT5G07990 | TRANSPARENT TESTA 7 (TT7) | 2.72 |
| AT4G09820 | TRANSPARENT TESTA 8 (TT8) | 50.52 |
| AT4G22880 | Leucoanthocyanidin dioxygenase (LDOX) | 4.75 |
| AT5G07990 | Cytochrome P450 (CYP706A6) | 2.72 |
| AT4G31970 | Cytochrome P450 (CYP82C2) | 3.46 |
| AT1G19630 | Cytochrome P450 (CYP722A1) | 2.06 |
| AT2G45550 | Cytochrome P450 (CYP76C4) | 1.72 |
| AT4G37430 | Cytochrome P450 (CYP91A2) | 1.84 |
| AT4G39510 | Cytochrome P450 (CYP96A12) | 1.51 |
| AT4G15393 | Cytochrome P450 (CYP702A5) | 1.51 |
| AT5G08250 | Cytochrome P450 (CYP86B1) | 1.58 |
| **Activated immune signaling pathway** | | |
| ***Polygalacturonase activity related protein*** | | |
| AT1G60590 | Pectin lyase-like superfamily protein (polygalacturonase activity) | 2.85 |
| AT2G36710 | Pectin lyase-like superfamily protein (polygalacturonase activity) | 2.57 |
| AT3G61490 | Pectin lyase-like superfamily protein (polygalacturonase activity) | 2.07 |
| AT1G02460 | Pectin lyase-like superfamily protein (polygalacturonase activity) | 1.97 |
| AT1G70500 | Pectin lyase-like superfamily protein (polygalacturonase activity) | 1.81 |
| AT1G10640 | Pectin lyase-like superfamily protein (polygalacturonase activity) | 1.76 |
| AT5G14650 | Pectin lyase-like superfamily protein (polygalacturonase activity) | 1.58 |
| AT5G06870 | Polygalacturonase inhibiting protein 2 (PGIP2) | 3.45 |
| ***Receptor-like kinases (RLKs) and receptor-like proteins(RLPs)*** | | |
| AT4G23220 | Cysteine-rich RLK (RECEPTOR-like protein kinase) 14 (CRK14) | 2.89 |
| AT5G60900 | Receptor-like protein kinase 1 (RLK1) | 3.12 |
| AT4G11890 | Protein kinase superfamily protein (RLK45) | 6.34 |
| AT3G23120 | Receptor like protein kinase 38 (RLP38) | 2.15 |
| AT1G21250 | Cell wall-associated kinase (WAK1) | 3.57 |
| AT1G21270 | Cell wall-associated kinase 2 (WAK2) | 1.89 |
| AT1G21230 | Cell wall associated kinase 5 (WAK5) | 2.68 |
| AT3G53590 | Leucine-rich repeat protein kinase family protein | 2.28 |
| AT1G49750 | Leucine-rich repeat (LRR) family protein | 1.62 |
| AT4G18250 | Receptor serine/threonine kinase, pathogenesis-related 5-like | 2.12 |
| AT1G11350 | Serine-threonine/tyrosine-protein kinase | 1.59 |
| \| ***Phytohormones synthesis and response-related proteins*** \| \| --- \| | | |
| AT2G19590 | ACC oxidase 1 (ACO1) | 1.59 |
| AT5G17220 | Glutathione S-transferase phi 12 (GSTF12) | 4.18 |
| AT2G29460 | Glutathione S-transferase tau 4 (GSTU4) | 1.59 |
| AT1G04370 | Pathogenesis-related transcriptional factor/ERF14 | 3.96 |
| AT5G47220 | Pathogenesis-related transcriptional factor/ERF2 | 1.57 |
| AT2G47520 | Pathogenesis-related transcriptional factor/ERF071 | 2.47 |
| AT4G28140 | Pathogenesis-related transcriptional factor/ERF054 | 3.46 |
| AT5G07310 | Pathogenesis-related transcriptional factor/ERF115 | 2.84 |
| AT5G51190 | Pathogenesis-related transcriptional factor/ERF105 | 1.5 |
| AT2G34600 | Jasmonate-zim-domain protein 7 (JAZ7) | 0.22 |
| AT1G30135 | Jasmonate-zim-domain protein 8 (JAZ8) | 0.28 |
| AT1G17420 | Lipoxygenase 3 (LOX3) | 0.18 |
| AT1G72520 | Lipoxygenase 4 (LOX4) | 0.52 |
| AT2G21900 | WRKY DNA-binding protein 59 (WRKY59) | 2 |
| AT2G40750 | WRKY DNA-binding protein 54 (WRKY54) | 1.54 |
| AT3G56400 | WRKY DNA-binding protein 70 (WRKY70) | 1.83 |
| AT5G64810 | WRKY DNA-binding protein 51 (WRKY51) | 1.95 |
| AT5G04230 | Phenyl alanine ammonia-lyase 3 (PAL3) | 1.63 |
| ***Pathogenesis-related protein proteins*** | | |
| AT4G33710 | CAP (Cysteine-rich secretory proteins, and Pathogenesis-related 1 protein) | 3.07 |
| AT4G33720 | CAP (Cysteine-rich secretory proteins, and Pathogenesis-related 1 protein) | 1.62 |
| AT3G57260 | Glycoside hydrolase, family 17, and Pathogenesis-related 2 protein | 5.27 |
| AT1G75040 | Pathogenesis-related thaumatin superfamily protein (PR5) | 11.53 |
| AT2G37130 | Peroxidase superfamily protein and PR9 | 1.75 |
| AT2G38540 | Lipid transfer protein 1 (LP1) and PR14 | 1.81 |
| AT5G55450 | Bifunctional inhibitor/lipid-transfer protein and PR14 family protein | 4.29 |
| AT5G39180 | RmlC-like cupins superfamily protein, Cupin 1, PR16 family protein | 5.31 |
| AT5G39150 | RmlC-like cupins superfamily protein, Cupin 1, PR16 family protein | 3.75 |
| AT5G61750 | RmlC-like cupins superfamily protein, Cupin 1, PR16 family protein | 2.45 |
| AT5G38910 | RmlC-like cupins superfamily protein, Cupin 1, PR16 family protein | 1.71 |
| AT5G39120 | RmlC-like cupins superfamily protein, Cupin 1, PR16 family protein | 1.66 |
| AT5G39110 | RmlC-like cupins superfamily protein, Cupin 1, PR16 family protein | 1.5 |
| The differentially expressed genes were with 1.5 fold change by comparing *GbCYP86A1-1* transgenic line and wild type in roots of 4-week old plants. Gene designations, descriptions of putatively encoded proteins and fold change were shown. | | |

**Table S6 Genes ontology (GO) analysis involved in biological processes from differential genes by comparing inoculated and un-inoculated *V. dahliae* in *GbCYP86A1-1* transgenic line.**

| GO_acc | Term type | Term | Query item | Bg item | P value | FDR |
| --- | --- | --- | --- | --- | --- | --- |
| GO:0009266 | P | response to temperature stimulus | 26 | [559](http://systemsbiology.cau.edu.cn/agriGOv2/termDetail.php?session=585255276.1&GO=GO:0009266) | 3.10E-11 | 2.10E-08 |
| GO:0006950 | P | response to stress | 63 | [3506](http://systemsbiology.cau.edu.cn/agriGOv2/termDetail.php?session=585255276.1&GO=GO:0006950) | 1.10E-07 | 3.10E-05 |
| GO:0050896 | P | response to stimulus | 92 | [6250](http://systemsbiology.cau.edu.cn/agriGOv2/termDetail.php?session=585255276.1&GO=GO:0050896) | 5.90E-07 | 9.10E-05 |
| GO:0042221 | P | response to chemical | 52 | [2853](http://systemsbiology.cau.edu.cn/agriGOv2/termDetail.php?session=585255276.1&GO=GO:0042221) | 1.30E-06 | 0.00016 |
| GO:0010035 | P | response to inorganic substance | 24 | [926](http://systemsbiology.cau.edu.cn/agriGOv2/termDetail.php?session=585255276.1&GO=GO:0010035) | 6.60E-06 | 0.00063 |
| GO:0006979 | P | response to oxidative stress | 16 | 453 | 6.70E-06 | 0.00063 |
| GO:0009628 | P | response to abiotic stimulus | 38 | 2022 | 2.20E-05 | 0.0019 |
| GO:0010033 | P | response to organic substance | 35 | 2023 | 0.00024 | 0.017 |
| GO:0009642 | P | response to light intensity | 7 | 144 | 0.00047 | 0.03 |
| GO:0009725 | P | response to hormone | 29 | 1631 | 0.00055 | 0.034 |
| GO:0009813 | P | flavonoid biosynthetic process | 9 | 93 | 3.90E-07 | 7.20E-05 |
| GO:0048511 | P | rhythmic process | 10 | 146 | 1.70E-06 | 0.00018 |
| GO:0046148 | P | pigment biosynthetic process | 8 | 148 | 9.20E-05 | 0.0068 |
| GO:0044711 | P | single-organism biosynthetic process | 30 | 1735 | 0.00069 | 0.036 |

**Table S7 Genes ontology (GO) analysis involved in biological processes from differential genes by comparing inoculated and un-inoculated *V. dahliae* in WT.**

| GO_acc | Term type | Term | Query item | Bg item | P value | FDR |
| --- | --- | --- | --- | --- | --- | --- |
| GO:0050896 | P | response to stimulus | [547](http://systemsbiology.cau.edu.cn/agriGOv2/termDetail.php?session=553670389.1&GO=GO:0050896) | 6250 | 6.80E-37 | 3.80E-33 |
| GO:0006950 | P | response to stress | [348](http://systemsbiology.cau.edu.cn/agriGOv2/termDetail.php?session=553670389.1&GO=GO:0006950) | 3506 | 1.40E-30 | 4.00E-27 |
| GO:0042221 | P | response to chemical | [269](http://systemsbiology.cau.edu.cn/agriGOv2/termDetail.php?session=553670389.1&GO=GO:0042221) | 2853 | 3.00E-20 | 3.40E-17 |
| GO:0006979 | P | response to oxidative stress | [80](http://systemsbiology.cau.edu.cn/agriGOv2/termDetail.php?session=553670389.1&GO=GO:0006979) | 453 | 7.60E-19 | 6.00E-16 |
| GO:0010033 | P | response to organic substance | [196](http://systemsbiology.cau.edu.cn/agriGOv2/termDetail.php?session=553670389.1&GO=GO:0010033) | 2023 | 8.80E-16 | 4.90E-13 |
| GO:0009628 | P | response to abiotic stimulus | [196](http://systemsbiology.cau.edu.cn/agriGOv2/termDetail.php?session=553670389.1&GO=GO:0009628) | 2022 | 8.40E-16 | 4.90E-13 |
| GO:0009725 | P | response to hormone | [148](http://systemsbiology.cau.edu.cn/agriGOv2/termDetail.php?session=553670389.1&GO=GO:0009725) | 1631 | 3.40E-10 | 8.50E-08 |
| GO:0009416 | P | response to light stimulus | [81](http://systemsbiology.cau.edu.cn/agriGOv2/termDetail.php?session=553670389.1&GO=GO:0009416) | 744 | 4.00E-09 | 9.40E-07 |
| GO:0009266 | P | response to temperature stimulus | [65](http://systemsbiology.cau.edu.cn/agriGOv2/termDetail.php?session=553670389.1&GO=GO:0009266) | 559 | 1.50E-08 | 2.80E-06 |
| GO:1901698 | P | response to nitrogen compound | [40](http://systemsbiology.cau.edu.cn/agriGOv2/termDetail.php?session=553670389.1&GO=GO:1901698) | 264 | 2.00E-08 | 3.30E-06 |
| GO:0010035 | P | response to inorganic substance | [92](http://systemsbiology.cau.edu.cn/agriGOv2/termDetail.php?session=553670389.1&GO=GO:0010035) | 926 | 2.10E-08 | 3.40E-06 |
| GO:0009409 | P | response to cold | [44](http://systemsbiology.cau.edu.cn/agriGOv2/termDetail.php?session=553670389.1&GO=GO:0009409) | 384 | 4.40E-06 | 0.00042 |
| GO:0009611 | P | response to wounding | [30](http://systemsbiology.cau.edu.cn/agriGOv2/termDetail.php?session=553670389.1&GO=GO:0009611) | 217 | 5.90E-06 | 0.00056 |
| GO:0006970 | P | response to osmotic stress | [59](http://systemsbiology.cau.edu.cn/agriGOv2/termDetail.php?session=553670389.1&GO=GO:0006970) | 636 | 4.50E-05 | 0.0031 |
| GO:0009414 | P | response to water deprivation | [38](http://systemsbiology.cau.edu.cn/agriGOv2/termDetail.php?session=553670389.1&GO=GO:0009414) | 347 | 4.70E-05 | 0.0032 |
| GO:0009651 | P | response to salt stress | [52](http://systemsbiology.cau.edu.cn/agriGOv2/termDetail.php?session=553670389.1&GO=GO:0009651) | 574 | 0.00021 | 0.011 |
| GO:0044699 | P | single-organism process | [681](http://systemsbiology.cau.edu.cn/agriGOv2/termDetail.php?session=553670389.1&GO=GO:0044699) | 9448 | 3.60E-23 | 6.80E-20 |
| GO:0019748 | P | secondary metabolic process | [71](http://systemsbiology.cau.edu.cn/agriGOv2/termDetail.php?session=553670389.1&GO=GO:0019748) | 492 | 5.60E-13 | 2.10E-10 |
| GO:0071555 | P | cell wall organization | [58](http://systemsbiology.cau.edu.cn/agriGOv2/termDetail.php?session=553670389.1&GO=GO:0071555) | 518 | 2.80E-07 | 3.80E-05 |
| GO:0055085 | P | transmembrane transport | [67](http://systemsbiology.cau.edu.cn/agriGOv2/termDetail.php?session=553670389.1&GO=GO:0055085) | 667 | 1.20E-06 | 0.00014 |
| GO:0006869 | P | lipid transport | [27](http://systemsbiology.cau.edu.cn/agriGOv2/termDetail.php?session=553670389.1&GO=GO:0006869) | 174 | 2.50E-06 | 0.00026 |
| GO:0016114 | P | terpenoid biosynthetic process | [20](http://systemsbiology.cau.edu.cn/agriGOv2/termDetail.php?session=553670389.1&GO=GO:0016114) | 120 | 2.00E-05 | 0.0015 |
| GO:0009698 | P | phenylpropanoid metabolic process | [24](http://systemsbiology.cau.edu.cn/agriGOv2/termDetail.php?session=553670389.1&GO=GO:0009698) | 164 | 2.10E-05 | 0.0016 |
| GO:0006811 | P | ion transport | [60](http://systemsbiology.cau.edu.cn/agriGOv2/termDetail.php?session=553670389.1&GO=GO:0006811) | 645 | 3.60E-05 | 0.0026 |
| GO:0009813 | P | flavonoid biosynthetic process | [16](http://systemsbiology.cau.edu.cn/agriGOv2/termDetail.php?session=553670389.1&GO=GO:0009813) | 93 | 9.30E-05 | 0.0056 |
| GO:0048878 | P | chemical homeostasis | [37](http://systemsbiology.cau.edu.cn/agriGOv2/termDetail.php?session=553670389.1&GO=GO:0048878) | 352 | 0.00013 | 0.0072 |
| GO:0046148 | P | pigment biosynthetic process | [19](http://systemsbiology.cau.edu.cn/agriGOv2/termDetail.php?session=553670389.1&GO=GO:0046148) | 148 | 0.00065 | 0.028 |
| GO:0006629 | P | lipid metabolic process | 76 | 994 | 0.0011 | 0.043 |
| GO:0009607 | P | response to biotic stimulus | [141](http://systemsbiology.cau.edu.cn/agriGOv2/termDetail.php?session=553670389.1&GO=GO:0009607) | 1253 | 4.50E-16 | 3.10E-13 |
| GO:0006952 | P | defense response | [153](http://systemsbiology.cau.edu.cn/agriGOv2/termDetail.php?session=553670389.1&GO=GO:0006952) | 1566 | 1.00E-12 | 3.40E-10 |
| GO:0042742 | P | defense response to bacterium | [49](http://systemsbiology.cau.edu.cn/agriGOv2/termDetail.php?session=553670389.1&GO=GO:0042742) | 354 | 7.40E-09 | 1.50E-06 |
| GO:0009620 | P | response to fungus | [57](http://systemsbiology.cau.edu.cn/agriGOv2/termDetail.php?session=553670389.1&GO=GO:0009620) | 573 | 9.90E-06 | 0.00084 |
| GO:0009627 | P | systemic acquired resistance | [14](http://systemsbiology.cau.edu.cn/agriGOv2/termDetail.php?session=553670389.1&GO=GO:0009627) | 67 | 4.10E-05 | 0.0029 |
| GO:0007165 | P | signal transduction | [141](http://systemsbiology.cau.edu.cn/agriGOv2/termDetail.php?session=553670389.1&GO=GO:0007165) | 1965 | 0.00015 | 0.0086 |
| GO:0009404 | P | toxin metabolic process | [12](http://systemsbiology.cau.edu.cn/agriGOv2/termDetail.php?session=553670389.1&GO=GO:0009404) | 60 | 0.0002 | 0.011 |
| GO:0010200 | P | response to chitin | [19](http://systemsbiology.cau.edu.cn/agriGOv2/termDetail.php?session=553670389.1&GO=GO:0010200) | 134 | 0.00021 | 0.011 |
| GO:0009636 | P | response to toxic substance | [15](http://systemsbiology.cau.edu.cn/agriGOv2/termDetail.php?session=553670389.1&GO=GO:0009636) | 102 | 0.00068 | 0.028 |
| GO:0009873 | P | ethylene-activated signaling pathway | [22](http://systemsbiology.cau.edu.cn/agriGOv2/termDetail.php?session=553670389.1&GO=GO:0071369) | 185 | 6.50E-04 | 0.028 |
| GO:0009751 | P | response to salicylic acid | [28](http://systemsbiology.cau.edu.cn/agriGOv2/termDetail.php?session=553670389.1&GO=GO:0009751) | 209 | 2.00E-05 | 0.0015 |

| **Table S8 Information on PCR primers used in this study.** | | |
| --- | --- | --- |
| Primer name | Sequence information (5′-3′) | Purpose |
| H2316F | CAGGGTGGAGCAGAAGATGTCTCT | *CYP86A1-1* for qRT-PCR |
| H2316R | GAGGTGGCAAATACAAAGTTAAGA |  |
| H2317F | GGAGCAAAAGATGTCTCTCACGC | *CYP86A1-2* for qRT-PCR |
| H2317R | TCCTCCATGCCTTTCCTTGTATCC |  |
| H2318F | CATGAAGCAAGGCCTTCGTGTTTA | *CYP86A1-3* for qRT-PCR |
| H2318R | CCACCAAAACCCCACTTGAACAA |  |
| H2320F | TCATTACAAACCGAAGACAAA | *CYP86B1-1* for qRT-PCR |
| H2320R | AATATCACGCAGGAACTTG |  |
| Y8991F | CGGTGGTGTGAAGAAGCCTCAT | Cotton histone3 (reference gene) |
| Y8991R | AATTTCACGAACAAGCCTCTGGAA |  |
| H2691F | TAAGTGGTCCAAGGGTATG | *GbCYP86A1-1* cloning |
| H2691R | GAGGTGGCAAATACAAAGT |  |
| H2692F | GATTTCGTGTAAATTACCTCAC | *GbCYP86A1-2* cloning |
| H2692R | TAAGCTACCGCATCCAATA |  |
| H2693F | AACGAACATTTCCCAACAACT | *GbCYP86A1-3* cloning |
| H2693R | TAAACCACCAAAACCCCACT |  |
| H3087F | GTGAGTAAGGTTACCGAATTCTGTCTCTCACACTGTTTATGAAGAAAGG | For constructing TRV: *GbCYP86A1-1* vector |
| H3087R | CGTGAGCTCGGTACCGGATCCCTAGTGTCTAAAACATGGAGAAATTCAA |  |
| H3088F | GTGAGTAAGGTTACCGAATTCAGAACGGCCTCCGAGTTTACTT | For constructing TRV: *GbCYP86A1-2* vector |
| H3088R | CGTGAGCTCGGTACCGGATCCAGCATATAGACACCCATATTATGTGATATG |  |
| H3089F | GTGAGTAAGGTTACCGAATTCTCTGTTCATGAAGCAAGGCCTT | For constructing TRV: *GbCYP86A1-3* vector |
| H3089R | CGTGAGCTCGGTACCGGATCCTTTATTTGTATAGCAAACAGACCACACA |  |
| H3091F | GTGAGTAAGGTTACCGAATTCGCCACCTATCAAACATGTACCATT | For constructing TRV: H3091 vector |
| H3091R | CGTGAGCTCGGTACCGGATCCTTGCCTGAGTGTCCTGGTCG |  |
| s4504F | ATTTACGAACGATAGGGTACCATGGAATTGGAAAACCTTCCATT | For constructing *GbCYP86A1-1*: GFP vector |
| s4504R | GCCCTTGCTCACCATGGATCCTGCAAGTAGACGCGGCTGC |  |
| s4507F | ATTTACGAACGATAGGGTACCATGGAAACCCTTCAATTAGTCTTCA | For constructing *GbCYP86A1-2*: GFP vector |
| s4507R | GCCCTTGCTCACCATGGATCCTGCAAGTGTACGAGGCTGCA |  |
| s4505F | ATTTACGAACGATAGGGTACCATGGAAACCCTTCCATTTGTCTT | For constructing *GbCYP86A1-3*:GFP vector |
| s4505R | GCCCTTGCTCACCATGGATCCTGGAAATTTGCGCGGCTG |  |
| S99F | GCTCTAGAATGGAATTGGAAAACCTTCC | For constructing pBI121: *GbCYP86A1-1* vector |
| S99R | CGGGATCCTGCAAGTAGACGCGGCTGCA |  |
| S100F | CCCAAGCTTATGGAAACCCTTCAATTAGT | For constructing pBI121: *GbCYP86A1-1* vector |
| S100R | GCTCTAGATGCAAGTGTACGAGGCTGCA |  |
| S101F | CCCAAGCTTATGGAAACCCTTCCATTTGT | For constructing pBI121: *GbCYP86A1-1* vector |
| S101R | GCTCTAGATGGAAATTTGCGCGGCTGCA |  |
| S3976F | CTCACGAGTTACCAGATAA | *GbCYP86A1-1* transgenic detection primer for qRT-PCR |
| S3976R | CAGTAGATCATCCGAAGG |  |
| S3979F | GAAGCCGACAGATTGATA | *GbCYP86A1-2* transgenic detection primer for qRT-PCR |
| S3979R | TAAGTCACCGTTGAGAAG |  |
| S3981F | ACTTGTTGCTTCGGTTAA | *GbCYP86A1-3* transgenic detection primer for qRT-PCR |
| S3981R | AATCTTCTCCAATCTCCATAG |  |
| S2777F | GACGCTTCATCTCGTCC | *Arabidopsis* Ubq5 (reference gene) |
| S2777R | CCACAGGTTGCGTTAG |  |
| ITS1-F | AAAGTTTTAATGGTTCGCTAAGA | *V. dahliae* (fungal biomass) |
| STVe1-R | CTTGGTCATTTAGAGGAAGTAA |  |
| GbPR1F | AAGAATGTGGGTTAGTGAGAGGGT | qRT-PCR for GbPR1 |
| GbPR1R | ACCACTTGAGTATAATGCCCGC |  |
| GbPR2F | CCACCAGCAGCAGAAGTTATCG | qRT-PCR for GbPR2 |
| GbPR2R | TTCAAGGTTTGCACTCGGAAGA |  |
| GbPR3F | ACTCCACAATCACCGAAGCCAT | qRT-PCR for GbPR3 |
| GbPR3R | GCATTCCAACCCTTACCACATTC |  |
| GbPR4F | TTGCGGCAATGGCTTCAATC | qRT-PCR for GbPR4 |
| GbPR4R | TGCTCTCACATTATTCGGCA |  |
| GbPR5F | GCCGTGATTCATACAGTTATCCTCA | qRT-PCR for GbPR5 |
| GbPR5R | TTGGCTCTTACTTCCGACCATCT |  |
| GbPR6F | CTGGGTGTCCTGGGAAGAAC | qRT-PCR for GbPR6 |
| GbPR6R | TTGTAGGGGGACGAACAACG |  |
| GbPR9F | CAACAGCGCCAACATACAGAG | qRT-PCR for GbPR9 |
| GbPR9R | CAGCACAAGAGACAATGCCAG |  |
| GbPR16F | CCCAAAGCTTGCCAAAGCA | qRT-PCR for GbPR16 |
| GbPR16R | GAACATTGGCTGGAGTGACC |  |
| AtPR5F | ACTTCACTCTAAGGAACAAT | For *Arabidopsis* transcriptome and qRT-PCR consistency test |
| AtPR5R | GTTACACATCTACCGTTTC |  |
| AtWAK1F | GTATCACCTGTAAGGAAGAT |  |
| AtWAK1R | GTAAAAGAACTGTCCTCCT |  |
| AtRPP4F | ATATACATGTGAACGTTTCTG |  |
| AtRPP4R | CTATATCAACATCAGCATCATC |  |
| AtDFRF | TTGAAGGTGTTGATGAGAA |  |
| AtDFRR | TGGATTGGTACGATAATGAA |  |
| AtERF054F | GAGCTAAATCTTTGTATTACGA |  |
| AtERF054R | TGATTCTGAAGTGTTGGAT |  |
| AtERF14F | CACTGCGGCTAATTCTTC |  |
| AtERF14R | CTCTTGCCCATGTTGATA |  |
| AtRLK1F | TCGTGGAATCTTGTATCTAC |  |
| AtRLK1R | GGATGTTAGTGAGCGTATA |  |
| AtCAPF | TATTGGCATTAGCTCTTGT |  |
| AtCAPR | CATAGTTCCAGGCGTATC |  |
| AtLOX3F | GAATCAATCCAGTGAACATT |  |
| AtLOX3R | GGTAATCCAACATATACAATCTA |  |
| AtZATTF | TTCTTTTCTACCGGAGAC |  |
| AtZATTR | TTAACTCCAAGAAATCGTTC |  |
| AtABCB22F | TTAGCGTAAGTATGCGAG |  |
| AtABCB22R | GAATTGAACCAGCAAGATT |  |
| AtLTP2F | AGCTGCAATAATGGTGAA |  |
| AtLTP2R | GCATTGTAGATTAGCAACTC |  |
| AtLTP3F | CATTACAATTCCACCTGTTG |  |
| AtLTP3R | ATAGGAGGAGTTGCGATA |  |
| AtLTP4F | GCTTCAAGAACCACAAAA |  |
| AtLTP4R | GGGTTTAGGGTTAGGTTT |  |
| AtPR2F | GTCTCAAGGAAGGTTCAG |  |
| AtPR2R | CGTTATCAACAGTGGACT |  |
| AtLTP1F | AAACCTCAACAGCATAGC |  |
| AtLTP1R | TAAGGAATATTGACTCCACAT |  |
| AtCRK14F | AAGTGTTGGTGACTATGAA |  |
| AtCRK14R | TCGCTGTATTGGTTACTG |  |
| AtERF071F | AACACTCAAGTAGAAGAAGAA |  |
| AtERF071R | CGGAATCTGATAGAATCTCA |  |
| AtERF2F | AATATAGAATCCGACTACGC |  |
| AtERF2R | CTCTTTCAATGGCAAACC |  |
| AtERF115F | CCGATGATGATGAGAACA |  |
| AtERF115R | CCTTGATCTTGAGTTGGA |  |
| AtWRKY59F | CAACATACGAAGGTAGACA |  |
| AtWRKY59R | AATATGGAGCAGAATGAGAG |  |
| AtWRKY70F | TTCAGTATCACATACATAGGAA |  |
| AtWRKY70R | ACGATGAGGATTGTCTTC |  |
| AtDIR1F | GATACATCAGTAGCGATAGAT |  |
| AtDIR1R | GAGTTCAGGATCAACACC |  |
| AtPAL3F | TCAACGACTTACTTGGTAG |  |
| AtPAL3R | TCATCAACATAGGAGAAGAC |  |
| AtABCG8F | AAGGAACTGTTGTGTACC |  |
| AtABCG8R | GGATTCTCGTAGTTCTTGA |  |
| AtCupinF | CTCTCATCCTGATAACCTTAT |  |
| AtCupinR | TTAGTGATTCCTGCTTGG |  |
| AtKCS6F | CCAAGTTCATCCCAATTC |  |
| AtKCS6R | CGGTTTGACATGTGTATG |  |
| AtWAK5F | GAGCAAGTTGTAGGTAGAA |  |
| AtWAK5R | TGGTATCCGAACAGTTATG |  |
| AtRLP38F | CCACAATCATCTACAAGGA |  |
| AtRLP38R | CTGGCTCTTCTAGTTCTG |  |
| AtWAK2 | TGAGTTCATTAACAGTGGTA |  |
| AtWAK2 | GGAATAGAAGCAGAAGAGT |  |
